# A metabolic, phylogenomic and environmental atlas of diatom plastid transporters from the model species *Phaeodactylum*

**DOI:** 10.3389/fpls.2022.950467

**Published:** 2022-09-22

**Authors:** Shun Liu, Mattia Storti, Giovanni Finazzi, Chris Bowler, Richard G. Dorrell

**Affiliations:** ^1^ Institut de Biologie de l’Ecole Normale Supérieure (IBENS), Ecole Normale Supérieure, Centre National De La Recherche Scientifique (CNRS), Institut National De La Santé Et De La Recherche Médicale (INSERM), Université Paris Sciences et Lettres (PSL), Paris, France; ^2^ CNRS Research Federation for the study of Global Ocean Systems Ecology and Evolution, FR2022/Tara Oceans GOSEE, 3 rue Michel-Ange, Paris, France; ^3^ Univ. Grenoble Alpes (UGA), Centre National Recherche Scientifique (CNRS), Commissariat Energie Atomique Energies Alternatives (CEA), Institut National Recherche Agriculture Alimentation Environnement (INRAE), Interdisciplinary Research Institute of Grenoble (IRIG), Laboratoire de Physiologie Cellulaire et Végétale (LPCV), Grenoble, France

**Keywords:** Bacillariophyta, metabolite import-export, plastid-targeted proteins, meta-genomics, *in silico* prediction, RNAseq, microarray, Protist

## Abstract

Diatoms are an important group of algae, contributing nearly 40% of total marine photosynthetic activity. However, the specific molecular agents and transporters underpinning the metabolic efficiency of the diatom plastid remain to be revealed. We performed *in silico* analyses of 70 predicted plastid transporters identified by genome-wide searches of *Phaeodactylum tricornutum*. We considered similarity with *Arabidopsis thaliana* plastid transporters, transcriptional co-regulation with genes encoding core plastid metabolic pathways and with genes encoded in the mitochondrial genomes, inferred evolutionary histories using single-gene phylogeny, and environmental expression trends using *Tara* Oceans meta-transcriptomics and meta-genomes data. Our data reveal diatoms conserve some of the ion, nucleotide and sugar plastid transporters associated with plants, such as non-specific triose phosphate transporters implicated in the transport of phosphorylated sugars, NTP/NDP and cation exchange transporters. However, our data also highlight the presence of diatom-specific transporter functions, such as carbon and amino acid transporters implicated in intricate plastid-mitochondria crosstalk events. These confirm previous observations that substrate non-specific triose phosphate transporters (TPT) may exist as principal transporters of phosphorylated sugars into and out of the diatom plastid, alongside suggesting probable agents of NTP exchange. Carbon and amino acid transport may be related to intricate metabolic plastid-mitochondria crosstalk. We additionally provide evidence from environmental meta-transcriptomic/meta- genomic data that plastid transporters may underpin diatom sensitivity to ocean warming, and identify a diatom plastid transporter (J43171) whose expression may be positively correlated with temperature.

## Introduction

Diatoms are one of the most abundant eukaryotic phytoplankton groups in the contemporary ocean (Benoiston et al., 2017; Vincent and Bowler, 2020), especially in nutrient-rich coastal upwelling regions and at high latitudes (Tréguer et al., 2017), and are responsible for 20% of total planetary net primary production (Nelson et al., 1995; Field et al., 1998; Vincent and Bowler, 2020). Understanding why diatoms have risen to a position of such unique ecological prominence is fundamental to understanding the function and future dynamics of the ocean ecosystem. Diatom plastids (*i.e.*, “chloroplasts”), as host organelles of photosynthesis (Marchand et al., 2018) and other central biosynthetic pathways (Terashima et al., 2011; Solymosi, 2012; Heydarizadeh et al., 2013; Dorrell et al., 2017; Dorrell et al., 2019; Nonoyama et al., 2019), play a critical role in marine primary production, and may be the key to explaining the dominance of diatoms in the ocean.

In contrast to the double membrane-surrounded plastids of primary endosymbiotic origin found in red algae, green algae and land plants, diatoms possess plastids which were acquired by a secondary (or higher) endosymbiosis event, in which a non-photosynthetic host eukaryote acquired a plastid by combining with a red algal endosymbiont (or one of its direct endosymbiotic descendants) (Bhattacharya et al., 2004; Dorrell and Bowler, 2017; Murik et al., 2019). As a result of their eukaryotic origins, diatom plastids are enclosed by four membrane layers (Bhattacharya et al., 2004; Pfeil et al., 2014). These membranes are, from the outside to inside: the plastid endoplasmic reticulum (cERM), the peri-plastidial membrane (PPM), the outer plastid membrane (OEM) and the inner envelope membrane (IEM) (Solymosi, 2012; Marchand et al., 2018). Diatom plastids possess thylakoid membranes inside the stroma (Pfeil et al., 2014), but these are arranged in a concentric “girdle lamella” around the stromal periphery, as opposed to the stacked and unstacked thylakoids found in plant plastids (Flori et al., 2017). The compartmental organization of the diatom plastid necessitates a diverse range of transporters to transport ions, substrates and products across its membranes (Marchand et al., 2018; Marchand et al., 2020). Although diatom plastids retain their own genomes, these are far smaller (< 150 genes) than their proteomes. Most of the approximately 2000 plastid-associated proteins) of diatoms are encoded in the nuclear genome, and transported into plastids following translation (Green, 2011; Gruber et al., 2015; Dorrell et al., 2017; Gruber and Kroth, 2017).

Previous phylogenomic studies of diatom genomes have revealed a chimeric origin of the diatom nucleus-encoded and plastid-targeted proteome (Oborník and Green, 2005; Nonoyama et al., 2019). These plastid-targeted proteins include not only proteins of red algal (i.e., endosymbiont) origin, but also proteins derived from green algae, bacteria, the host cell, and even a large number of proteins specific to this lineage without obvious homologues elsewhere in the tree of life (Dorrell et al., 2017; Nonoyama et al., 2019). This diverse combination of genes from different origins has also contributed to metabolic innovations in the diatom plastid distinctive from those of plants (Marchand et al., 2018; Nonoyama et al., 2019). These include a complete plastid-targeted ornithine cycle, which interacts with a complete mitochondria-targeted urea cycle in diatom amino acid metabolism and recycling (Allen et al., 2011; Nonoyama et al., 2019); a complex suite of plastid-targeted proteins involved in iron storage, acquisition, and stress tolerance (Gao et al., 2021); and elaborate CO_2_ concentration and carbon metabolism systems (e.g., a complete glycolysis-gluconeogenesis pathway) not known in plant plastids (Marchand et al., 2018; Nonoyama et al., 2019). Moreover, many of these diatom-associated plastid metabolic innovations, including but not limited to central nitrogen and carbon metabolism, depend on intricate crosstalk between diatom plastids and mitochondria, which typically show close proximity to one another in diatom cells (Prihoda et al., 2012; Bailleul et al., 2015; Uwizeye et al., 2020). These plastid-related novel metabolic activities may depend on transporter innovations across the four membranes, or metabolite exchanges between organelles (Shai et al., 2016; Dorrell et al., 2017; Mix et al., 2018).

Although previous studies have focused on diatom plastid metabolic innovations (Bailleul et al., 2015; Marchand et al., 2018; Nonoyama et al., 2019; Gao et al., 2021), little is known specifically concerning their plastid transporter diversity, molecular and environmental functions. Marchand et al. presented overviews of the localization and activity of ion and metabolite plastid transporters from algae to land plants (Marchand et al., 2018; Marchand et al., 2020); and Brownlee et al. recently summarized key mechanisms of diatom key nutrient transport and acquisition (Brownlee et al., 2022). Specific studies have further elaborated on the diversity of diatom plastid sugar transporters (which typically transport triose phosphates) (Moog et al., 2015; Moog et al., 2020); low CO_2_ induced $HCO_{3}^{-}$ transporters (SLC) (Nakajima et al., 2013; Matsuda et al., 2017; Tsuji et al., 2017); nitrate/peptide transporters (Santin et al., 2021); and nucleotide triphosphate transporters (Ast et al., 2009).

Finally, diatoms and their plastid metabolism may act as important bellwethers of environmental and climate change (Spetea et al., 2014). Previous studies have demonstrated that both geochemical factors (e.g., iron and copper concentrations) (Hippmann et al., 2020; Kong and Price, 2021; Turnšek et al., 2021) alongside physical factors (pH, CO_2_ availability, and temperature) (Tong et al., 2021; Zhong et al., 2021) directly influence diatom photosynthetic activity and abundance. Understanding the roles of specific plastid transporters in these responses, such as the production and accumulation of plastid metabolites and compatible ions implicated in stress tolerance (Marchand et al., 2018), and intracellular communication between various subcellular compartments may allow more nuanced prediction of diatom responses to dynamically changing environments (Murik et al., 2019; Hippmann et al., 2020).

In this study, we use bioinformatic techniques to profile the predicted functions of 70 plastid-targeted transporters inferred from the well-characterized genome of the model diatom *Phaeodactylum tricornutum*, with complete models realised for > 99% of its probable encoded genes *via* transcriptomic and proteogenomic reannotations (Bowler et al., 2008; Rastogi et al., 2018; Yang et al., 2018); alongside extensive gene expression and epigenomic resources to understand its encoded functions (Veluchamy et al., 2013; Veluchamy et al., 2015; Ashworth et al., 2016; Ait-Mohamed et al., 2020; Zhao et al., 2020). Considering *Phaeodactylum*-specific gene expression trends, phylogenetic similarity to transporters from other organisms, and the broader environmental expression dynamics of homologues identified within the *Tara* Oceans survey (Villar et al., 2018), we profile the probable biological, metabolic and eco-physiological diversity of the diatom plastid transporter repertoire, and identify new candidate transporters for diatom-specific plastid metabolic activities and environmental resilience. Our study is the first systematic exploration of plastid transporter diversity across diatom algae.

## Materials and methods

### Comparison of plastid transporters from *Arabidopsis thaliana* and *Phaeodactylum tricornutum*

A list of 77 A*. thaliana* plastid transporters (**Supplementary Table S1**) was obtained from ChloroKB (Gloaguen et al., 2017; Gloaguen et al., 2021) and integrated with other characterized inorganic ion transporters from the literature (Finazzi et al., 2015; Marchand et al., 2018). Transporter gene functions were determined from the literature (Finazzi et al., 2015; Marchand et al., 2018), as integrated into TAIR (accessed 06/2021) (Huala et al., 2001).

A list of 70 plastid transporter genes (**Supplementary Table S2**) was found in the version 3 annotation of the *P. tricornutum* genome (Bowler et al., 2008; Yang et al., 2018). These transporters were identified through the presence of plastid-targeting sequences, inferred *in silico* using ASAFind version 2.0 (Gruber et al., 2015) with SignalP 3.0 (Bendtsen et al., 2004) and HECTAR (Gschloessl et al., 2008), run under default conditions; and the presence of transporter functions inferred annotated by KEGG (using BLASTkoala) (Kanehisa et al., 2016), Gene Ontology Annotation (Harris et al., 2004) or PFAM (using InterProScan) (Mulder and Apweiler, 2007).

BLASTp v 2.12.0 (Altschul et al., 1990; Hernández-Salmerón and Moreno-Hagelsieb, 2020) was used to find potential homologues of candidate *P. tricornutum* plastid transporters in the predicted protein models encoded by the complete genome of *A. thaliana* with threshold percentage identity ≥ 30% and E-value ≤ 1e-05, and vice-versa. The localization of *P. tricornutum* homologues of *A. thaliana* transporters was inferred based on *in silico* prediction, using ASAFind and HECTAR as before; MitoFates, using threshold value 0.35 (Fukasawa et al., 2015); and WolfPSort (Horton et al., 2006), using the consensus prediction of animal, fungi and plant models. Homologue localizations were divided into mitochondrial (M), plastid (P), plasma membrane or endoplasmic reticulum (PM/ER), and homologues with unclear localizations (e.g., N-incomplete gene annotations) were listed as undefined (U). The localizations of *A. thaliana* homologues of *P. tricornutum* plastid transporters, as inferred by BLASTp, were based on experimental data (https://suba.plantenergy.uwa.edu.au/; https://www.rostlab.org/services/locDB/) (Rastogi and Rost, 2011; Hooper et al., 2017), and when not available inferred from WolfPSORT (https://wolfpsort.hgc.jp/) using a plant reference dataset (Horton et al., 2006). Localizations were sorted as mitochondria (M), plastid (C), Vacuole (V), Golgi (G) and plasma membrane (PM).

*A. thaliana* plastid transporters were searched by SUBA (https://suba.live/), and manually checked in the literature if not cataloged. For *P. tricornutum*, plastid transporters that have been experimentally verified by GFP were identified from the literature (Gruber et al., 2015; Moog et al., 2015; Dell'Aquila et al., 2020), alongside *P. tricornutum* homologues (identified by genome-to-genome RbH) to plastid transporters experimentally identified from the purified plastid proteome of the diatom *Thalassiosira pseudonana* (Schober et al., 2019). All 67 A*. thaliana* plastid transporters and 7 P*. tricornutum* plastid transporters that have been experimentally verified (GFP localization, plastid proteome, mass spectroscopy) are noted in **Supplementary Tables S1**, **S2**.

### Identification of metabolic pathways correlated with *P. tricornutum* plastid transporters

The complete version 3 annotation of the *P. tricornutum* genome (Bowler et al., 2008; Yang et al., 2018) was filtered for genes showing probable linked functions to each of the 70 plastid transporters, using a composite approach based on seven filtered conditions to generate a composite “score” for probable pathway linkage to each transporter. Four of these conditions related to gene co-regulation patterns: (i) the repartition of each gene into WGCNA (weighted genome correlation network analyses) modules in a previous meta-study of *P. tricornutum* RNAseq data (Ait-Mohamed et al., 2020), with genes that were retrieved in the same module as the query transporter scored as +1; and the crude Pearson Correlation coefficient of each genes to each the query transporter calculated in (ii) *P. tricornutum* RNA-seq data (Ait-Mohamed et al., 2020), (iii) *P. tricornutum* microarray data integrated into DiatomPortal (http://networks.systemsbiology.net/diatom-portal/) (Ashworth et al., 2016), and (iv) the *Thalassiosira pseudonana* homologues (as defined by RbH search) of *P. tricornutum* genes in *T. pseudonana* microarray data (Ashworth et al., 2016), with genes retrieving any correlation coefficient > 0.5 scored as +1. In addition, three conditions relating to localization and evolution were considered: (v) the consensus *in silico* targeting predictions inferred with ASAFind and HECTAR (Gschloessl et al., 2008; Gruber et al., 2015), with proteins showing chloroplast targeting predictions scored as +1; and (vi) the inferred origin point and (vii) pattern of inferred loss of each transporter across the ochrophytes in a previously published RbH study (Nonoyama et al., 2019), with only genes that showed the same pattern of origin as the query gene (i.e., earliest ochrophyte common ancestor for which the presence of gene homologues could be inferred) or loss (i.e., monophyletic ochrophyte clades descended from the inferred common ancestor of the given gene in which the gene was not detected) scored as +1. Genes in the *P. tricornutum* genome for which a composite score of at least three was calculated were deemed to be associated to each query transporter (**Supplementary Table S3**).

Plastid metabolic pathways were annotated from previously constructed datasets (Nonoyama et al., 2019; Ait-Mohamed et al., 2020), PFAM, KEGG and KOG. Pathways for which more than three genes recovered a score ≥ +3 to a given transporter were considered to show a functional association with the transporter gene.

### Correlation calculations of *P. tricornutum* plastid transporters with mitochondria-encoded genes

Pearson correlation coefficients were calculated using DiatomPortal microarray values for 34 genes in the *P. tricornutum* mitochondrial genome (Oudot-Le Secq et al., 2007; Ashworth et al., 2016), and 66 P*. tricornutum* plastid transporter genes for which corresponding microarray data were available. Microarray values were drawn from studies of light: dark acclimation dynamics in 17 different conditional processing groups (Nymark et al., 2009; Nymark et al., 2013; Valle et al., 2014); and the average of the correlation coefficients observed for each gene in the mitochondrial genomes was calculated for each transporter gene. Equivalent calculations were not performed for RNAseq data (Ait-Mohamed et al., 2020) due to the exclusion of mitochondrial transcripts from polyA-selected RNA libraries; or from *Thalassiosira* equivalents of *Phaeodactylum* genes due to the absence of generated mitochondrial microarray probes for this species (Ashworth et al., 2016).

### Evolutionary conservation of *P. tricornutum* plastid transporters

70 plastid transporters were searched by RbH against 287 genomes and transcriptomes belonging to 11 taxonomic groups across the stramenopiles (diatoms, ochrophytes, and aplastidic groups), cryptomonads and haptophytes (**Supplementary Table S4**), with threshold E-value< 1e-05 and percentage identity ≥ 30%. *In silico* targeting predictions were performed for each transporter using ASAFind, HECTAR and MitoFates as defined above. The deeper evolutionary history of each transporter was summarized from previously published data (Dorrell et al., 2021), considering horizontal, endosymbiosis-associated and endosymbiotic gene transfers into and out of the diatom and ochrophyte common ancestors, from both prokaryotic and eukaryotic sources.

### Calculation of environmental expression trends associated with *P. tricornutum* plastid transporters

The relationships between *P. tricornutum* plastid transporters and environmental factors were analyzed by correlating the relative abundances of transporter homologous sequences to environmental factors from *Tara* Oceans Version 1 (Carradec et al., 2018) on the Ocean Gene Atlas (OGA) website (http://tara-oceans.mio.osupytheas.fr/ocean-gene-atlas/) (Villar et al., 2018). The FASTA-formatted protein sequence of each plastid transporter was searched on the OGA portal by BLASTp with threshold E-value 1x10^-05^. Gene abundance estimates were calculated from the meta-genomes (MetaG) database, and transcript abundance estimates were calculated from the meta-transcriptomes (MetaT) database. Complete details of homologue sequences, taxonomic annotations, and environmental distributions of each diatom transporter meta-gene, as inferred by Ocean Gene Atlas website, are provided in the linked osf.io repository https://osf.io/89vm3/ in the folder “Transporters”

The resulting raw homologues downloaded from OGA website were filtered to only retrieve those homologue sequences to diatom plastid-targeted transporters. First, a reference dataset was constructed for each gene from all plastid-targeted transporters identified in stramenopile, haptophyte and cryptomonad libraries as defined above, and the best-matching sequence identified from 151 non-redundant taxonomic categories from a previously constructed whole-tree of life dataset (Dorrell et al., 2021), inferred by BLASTp using the query with threshold e-value 10^-05^. The *Tara* Oceans sequences were searched against the reference dataset by BLASTp with the –max_target_seqs 1 threshold applied, and only sequences with diatom plastid-targeted best hits were retained. A second BLASTp was performed against the complete protein sequence annotation models from the *P. tricornutum* genome using the same criteria, and only sequences that obtained best hits with the specific queried gene ID were retained. The intersection of each set of retained genes were selected and combined with the reference dataset, and aligned using MAFFT (Katoh et al., 2002) with the –auto setting. Poorly aligned sequences were manually removed, defined visually by <50% overlap with the conserved domain region of the query transporter, and a guide phylogenetic tree was generated for each curated alignment using the NJ tree-building function in Geneious v 10.0.9 (Kearse et al., 2012) and 100 replicates. Finally, Tara meta-genes corresponding to diatom plastid transporters in the NJ tree topology, to the exclusion of all other cultured homologues, were extracted for 65 plastid transporter families, and used for quantitative analysis (**Supplementary Table S5**). Complete fasta files of *Tara* Oceans homologues for each transporter, along with raw and curated alignments and tree topologies are provided in the linked osf supporting database https://osf.io/89vm3/ in the folder “Transporters”.

MetaT and MetaG abundances of verified transporter homologues were calculated for all stations, all size fragments (0.8-5, 5-20, 20-180 and 180-2000μm) and different depths (DCM: deep chlorophyll maximum layer; SRF: upper layer zone). Absolute correlation coefficients were calculated by Pearson correlation analysis between the sum MetaT total relative abundance, the sum MetaG total relative abundance (with the exception of transporter J12788, for which no MetaG data were available), and measured environmental factors for each station, following data from PANGAEA (Pesant et al., 2015). A two-tailed *t*-test was used to determine if the MetaT-MetaG total was significantly positively or negative correlated to each environmental variable (**Supplementary Table S5**). P-values were shown as positive if $R_{metaT}^{2}>\;R_{metaG}^{2}$ , and negative value if $R_{metaT}^{2}>\;R_{metaG}^{2}$ ; with significance threshold P= 0.05.

Finally, environmental parameters which were shown to be strongly positively or negatively correlated in MetaT compared to MetaG data (MetaT-MetaG) in at least three of the ten combinations of different size fractions and depth conditions (DCM<0.8; DCM 0.8-5; DCM 5-20; DCM 20-180; DCM 180-2000; SRF<0.8; SRF 0.8-5; SRF 5-20; SRF 20-180; SRF 180-2000), were inferred to be concluded to significantly related to the expression of the transporter. To allow a more intuitive view of the results, five pigment concentration parameters related to photosynthetic potential (Liu et al., 2009; Puissant et al., 2021); and net primary production, particulate inorganic, organic and total carbon, previously used as metrics for modeling carbon export from the ocean surface layer, were merged together as primary production-related categories (Guidi et al., 2016; Leblanc et al., 2018). A threshold of significant (P< 0.05) correlations in ≥ 3 depth and size fractions combinations for ≥ 8 of the ten selected parameters across this merged dataset was deemed to correspond to a significant relationship with primary production.

## Results

### Limited conservation of plastid transporters between *Arabidopsis thaliana* and *P. tricornutum*

To gain an overall appreciation of the similarity of the plastid transporters in diatoms to those of the more well-studied but less complex plastids of higher plants, the total plastid transporter proteomes of *A. thaliana* and *P. tricornutum* were compared using reciprocal BLASTp-BLASTp searches. A total of 77 A*. thaliana* plastid transporters were defined from the literature (Finazzi et al., 2015; Marchand et al., 2018), ChloroKB (Gloaguen et al., 2017), and NCBI (Wheeler et al., 2008) (**Supplementary Table S1**), whereas 70 predicted *P. tricornutum* plastid transporters were found based on (i) the presence of a transport-associated KEGG, GO or PFAM domain (Rastogi et al., 2018; Ait-Mohamed et al., 2020), and (ii) the presence of a plastid targeting sequence inferred by *in silico* prediction with ASAFind (Gruber et al., 2015) and HECTAR (Gschloessl et al., 2008) from the version 3 genome annotation (**Supplementary Table S2**). The 70 inferred *in silico Phaeodactylum* transporters include the small number of plastid transporters that have been experimentally verified by GFP localization or mass spectroscopy in diatoms (**Supplementary Tables S1**, **S2**), whereas only ten of the *A. thaliana* transporter localizations are still putative and have yet to be formally experimentally confirmed (**Supplementary Table S1**).

**Figure 1:**
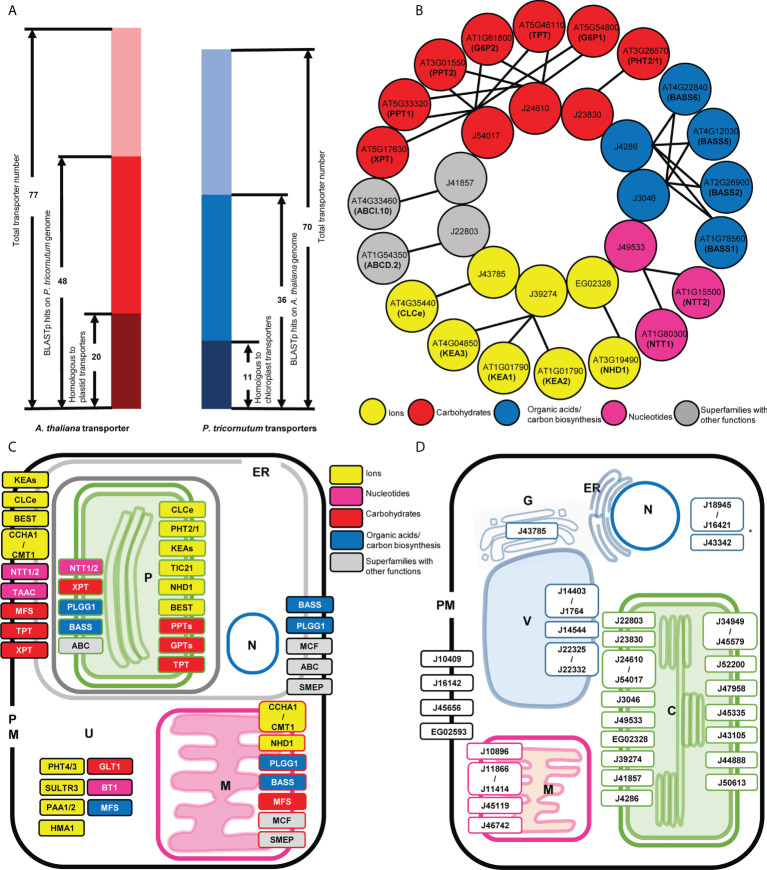
Comparison of *thaliana* and *P. tricornutum* plastid transporters. **(A)** The number of BLASTp homologues identified between 77 A*. thaliana* plastid transporters and 70 P*. tricornutum* plastid transporters. Dark red and blue represent transporters that had BLASTp homologue annotated as transporters within the searched genome; red and blue represent the number of plastid transporters that had BLASTp homologues (e-value < 1E-5; identity >30%) not annotated as transporters within the searched genome; and light red and light blue represent the number of transporters that do not have identifiable BLASTp homologues in the searched genome. **(B)** Transporter BLASTp equivalent matches in detail. One-to-one and one-to-many correspondences are indicated by arrow lines; circle colors represent functional categories, with grey indicating transporter superfamilies that have different functions and thus have uncertain function. **(C)** Predicted localization of *thaliana* plastid transporter homologues in *P. tricornutum*. Transporters are named according to the common abbreviation/gene family name in *thaliana*, and their localization in *P. tricornutum* are shown according to homologue predictions from ASAFind, HECTAR and MitoFates, ER, endoplasmic reticulum, P, plastid, N, nucleus M, mitochondria, PM, plasma membrane, U, unknown/undefined. Colored and grey boxes represent as above. Multiple localisations indicate transporters for which *P. tricornutum* homologues were inferred to localise to more than one organelle. **(D)** Predicted localization of *P. tricornutum* plastid transporter homologues in *thaliana*. Transporters are named according to the gene ID in the version 3 *P. tricornutum* genome annotation, and their localization in *thaliana* are shown according to experimental data (https://suba.plantenergy.uwa.edu.au/ or https://www.rostlab.org/services/locDB/), or when not available inferred from WolfPSORT, C, plastid, ER, endoplasmic reticulum, G, Golgi, M, mitochondria, PM, plasma membrane, V, Vacuole, N, nucleus, *, undefined targeting.

48 *A. thaliana* transporters, from a diverse range of families, were found to have possible homologues (BLASTp: E-value ≤ 1e-05, identity ≥ 30%) in the *P. tricornutum* proteome, but only 20 of these proteins have at least one homologue that was predicted to contain plastid targeting sequences in *P. tricornutum* using ASAFind or HECTAR as above (**Figure 1A**; **Supplementary Table S1**). In turn, while 36 of the *P. tricornutum* plastid transporters had probable BLASTp homologues in the *A. thaliana* genome, only 11 of these have homologues previously annotated as transporters in the *A. thaliana* plastid proteome (**Figure 1A**, **Supplementary Table S1**). Our understanding of which transporters are plastid-targeted in *P. tricornutum* depends largely on the quality of genome annotation in the absence of an experimentally resolved purified plastid proteome, although the version 3 annotation used is inferred to be largely N-terminally correct considering both transcriptomic and proteomic data (Rastogi et al., 2018; Schober et al., 2018; Yang et al., 2018).

The 20 A*. thaliana* plastid transporters that were found to correspond directly to 11 P*. tricornutum* plastid transporters, matched with both one-to-one and one-to-many correspondences (**Figures 1A, B**, **Supplementary Tables S1**, **S2**). These one-to-many correspondences probably reflect independent duplications of genes encoding plastid transporters in both the *A. thaliana* and *P. tricornutum* genomes (Jaillon et al., 2007; Parks et al., 2018). The homologue equivalent transporters found between *A. thaliana* and *P. tricornutum* perform key plastid functions, including the nucleotide transporter family (NTT1/2, matching *Phaeodactylum* transporter J49533) essential for the import of NTPs from the cytoplasm into plastid (Ast et al., 2009) (**Figure 1B**). Three plastid transporters belonging to the AtKEA family (KEA1, KEA2, KEA3) that function as K^+^/H^+^ antiporters and play essential roles in plastid development, ion homeostasis, and photosynthesis (DeTar et al., 2021), correspond to one *P. tricornutum* plastid transporter J39274. Four members of the BASS family (Bile acid/sodium symporter-like transporter; BASS1, BASS2, BASS5, BASS6) which exhibit a wide range of substrate specificities, including non-bile acid organic compounds such as pyruvate, steroids, methionine-derived glucosinolates (GSL) and exogenous substances (South et al., 2017), showed equivalence to one sodium/bile acid transporter (J4286) and one sodium/pyruvate transporter (J3046) in the *P. tricornutum* genome (**Figure 1B**).

Two annotated *P. tricornutum* triose phosphate transporters (J24610, J54017) retrieved BLASTp results with the *A. thaliana* TPT, glucose 6-phosphate transporter (GPT), xylulose 5-phosphate transporter (XPT), and phosphoenolpyruvate transporters (PPT; **Figure 1B**). This latter result supported a recent study of plastid phosphate transporters in the non-photosynthetic diatom *Nitzschia* sp. NIES-3581, which suggests that TPT transporters are capable of transporting multiple metabolites including triose phosphates (TPs) and phosphoenolpyruvate (PEP) (Moog et al., 2020), whereas no recognizable hexose phosphate transport system is associated with diatom plastids (Moog et al., 2015). An ABC supergroup transporter ABCI.10 (AT4G33460) putatively involved in metal homeostasis retrieved the *P. tricornutum* plastid transporter J41857 as the equivalent (**Figure 1B**).

The RbH results provide insights into the putative functions of specific *P. tricornutum* transporters that have not previously been annotated. These include J39274 which was annotated within the version 3 P*. tricornutum* genome as an Na^+^/H^+^ transporter by CDD search and PFAM; but in our search shows more similarity to KEA family transporters. We note that J39274 has recently and independently been functionally characterised as the diatom KEA3 (Seydoux et al., 2022). We further identify a phosphate permease J23830, which was found to show homology to the *A. thaliana* phosphate transporter PHT2/1, previously believed to be restricted to green and red algae and not found elsewhere in the tree of life (Pfeil et al., 2014; Marchand et al., 2018). Other key *A. thaliana* plastid transporters were either not detected, or only possess non-plastid targeted homologues in *P. tricornutum* such as the SO4^2-^/H^+^ antiporter superfamily SULTR (Cao et al., 2013) and metal transporter P-type ATPases (PAA1, PAA2) (Hanikenne and Baurain, 2013).

### Differential localizations of diatom homologues of plant plastid transporters

Over their evolutionary history, proteins can undergo re-localization: proteins currently targeted to the plastid may have originally been recruited from other compartments inside the host cell (Liu et al., 2014), while plastid-targeted proteins may also be relocated to support other host organelles (Martin et al., 2002; Dorrell et al., 2019). This phenomenon may have occurred with *A. thaliana* and *P. tricornutum* plastid transporters, where some homologues (BLASTp: E-value ≤ 1e-05, identity ≥ 30%) apparently displayed a different localization in each species (**Figure 1A**; **Supplementary Table S1**, **S2**). Based on *in silico* localization predictions and experimental data, the predicted localizations of the *P. tricornutum* homologous sequences of *A. thaliana* plastid transporters were mapped in the *P. tricornutum* cell. These were found to be distributed across the plastid (P), mitochondria (M), plasma membrane (PM) and endoplasmic reticulum (ER) (**Figure 1C**; **Supplementary Table S1**).

Previous studies of plastid localizations have been inferred experimentally for certain TPTs (J24610 and J54017) (2005; Kilian and Kroth, 2004; Moog et al., 2015) and NTT1 (J49533) (Ast et al., 2009) in *P. tricornutum*, coherent with *in silico* predictions. Moreover, by reciprocal BLAST best hit (RbH), six *P. tricornutum* plastid transporters (a probable Na^+^/H^+^ antiporter family protein EG02328, Tic110 J50540, TPT J24610, a formate/nitrite transporter J13076, NTT1 J49533, and a sodium/pyruvate transporter J3046) were found to be homologous to plastid transporters (**Supplementary Table S2**) identified in an experimental plastid proteome study of the diatom *Thalassiosira pseudonana* (Schober et al., 2019), The closest *P. tricornutum* homologues of the *A. thaliana* glucose-6-phosphate and phosphate transporters (PHT2/1, PPTs, GPTs: AT3G26570, AT5G33320 and AT3G01550, AT5G54800 and AT1G61800) (**Supplementary Table S2**) were further inferred to have exclusively plastid localizations by *in silico* prediction (**Figure 1C**), although as discussed above these homologues may be triose phosphate transporters (Moog et al., 2015; Moog et al., 2020).

Other *P. tricornutum* homologues of *A. thaliana* transporters did not show exclusive plastid localization, but were also inferred to localize to the PM/ER (**Figure 1C**), such as probable homologues of the KEA K^+^/H^+^ antiporters family (AT1G01790, AT4G04850 and AT1G01790). The *P. tricornutum* homologues of a thylakoid ADP/ATP carrier protein (AT5G01500), implicated in both ATP/ADP and 3’-phosphoadenosine 5’-phosphosulfate (PAPS) transport across the *A. thaliana* plastid envelope (Thuswaldner et al., 2007; Gigolashvili et al., 2012), were only predicted to be localized to the *P. tricornutum* PM/ER (**Figure 1C**). At least some of these transporters may localize to the *P. tricornutum* cERM and therefore still be implicated in metabolite exchange with the *P. tricornutum* plastid. In the case of the *P. tricornutum* PAPS transporter, a complete plastid cysteine synthesis pathway from PAPS reductase to cysteine synthase is known for diatoms, but no plastid-targeted adenosine sulphate kinase is known, indicating PAPS is likely to be imported from the cytoplasm across the cERM (Dorrell et al., 2017). Finally, we note that one putative phosphate permease (J23830) in our dataset has previously been GFP localized to the *P. tricornutum* cERM, and not the plastid (Dell'Aquila et al., 2020). It remains to be determined if this discrepancy relates to a possible dual-localisation or mis-targeting considering either overexpression or *in silico* prediction.

Moreover, some *P. tricornutum* homologues of *A. thaliana* plastid transporters showed mitochondrial localization predictions (**Figure 1C**), e.g., homologues of glycolate/glycerate translocator 1 (PLGG1; AT1G32080) (Pick et al., 2013), the GDT1-family Ca^2+^/H^+^ antiporter CCHA1 and CMT1 (AT1G64150 and AT4G13590) (Wang et al., 2016), and members of the BASS superfamily (AT2G26900, AT4G22840, AT4G12030, AT1G78560) (**Figure 1C**). These may represent distant mitochondrial homologues of *A. thaliana* plastid transporters, with either plastid, mitochondrial, or alternative ancestral localizations.

The subcellular locations of the *A. thaliana* homologues of 36 P*. tricornutum* plastid transporters included the plastid (C), but also the plasma membrane (PM), mitochondria (M), Golgi body (G) and vacuole (V) (**Figure 1D**, **Supplementary Table S2**).

Five *P. tricornutum* transporters were inferred to possess dual plastid/mitochondria-targeting sequences by *in silico* prediction (Gile et al., 2015; Dorrell et al., 2017), i.e., a plastid-targeting prediction with either ASAFind or HECTAR, and a mitochondria-targeting prediction with either HECTAR or MitoFates. These proteins were: a probable Na^+^/H^+^ antiporter family protein (EG02328); a transporter of unknown function (EG02514); a multidrug and toxic compound extrusion family/MATE efflux family protein (J35587); a glycolipid transporter (J36726); and a sodium/bile acid transporter (J4286) (**Supplementary Table S2**), suggesting more intricate metabolic interactions between both organelles in the diatom cell.

### Transcriptional co-regulation reveals metabolic pathway linkages of *P. tricornutum* plastid transporters

To classify putative roles for the diverse range of transporters associated with the *P. tricornutum* plastid with no clear homologues in *A. thaliana*, we inferred probable biochemical functions for each transporter. Large-scale microarray and RNA sequencing data (Ashworth et al., 2016; Ait-Mohamed et al., 2020); alongside other annotations such as inferred evolutionary origin and loss (Nonoyama et al., 2019; see Materials and Methods) were availed to identify metabolic pathways associated with each plastid transporter in the *P. tricornutum* genome (**Supplementary Table S3**).

**Figure 2:**
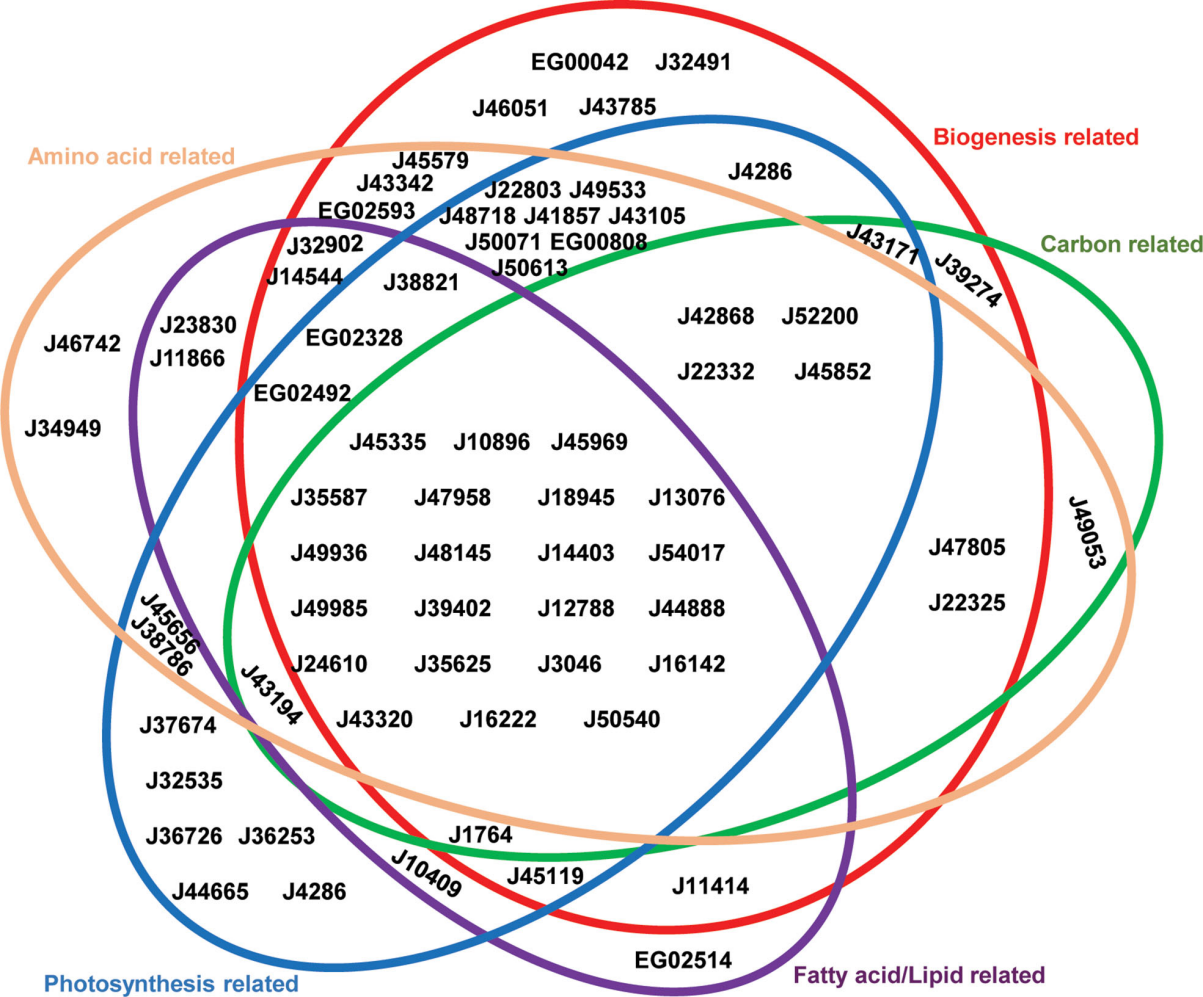
Venn Diagram showing associated pathways of *P. tricornutum* plastid transporters. Plastid-transporter associated pathways were identified from a set of associated genes in the *P. tricornutum* genome, considering seven co-regulation, evolutionary and localization conditions (details in Materials and Methods). Each metabolic pathway annotated from PFAM, KEGG, KOGG, was manually merged into five main categories, represented as different colored circles. These are: photosynthesis-related = photosynthetic and light-harvesting proteins, enzymes involved in chlorophyll and carotenoid biosynthesis, and mitochondrial respiration-related pathways; biogenesis-related = protein subunits of the plastid ribosome, protein import, division, DNA transcription and replication machineries; carbon-related = enzymes with KOGG annotations related to carbohydrate transport and metabolism; fatty acid/lipid-related = enzymes with KOGG annotations related to fatty acid or lipid metabolism; and amino acid-related = enzymes with KOGG annotations related to amino acid transport and metabolism, tRNA activation and Fe-S cluster synthesis. Transporters are shown as gene IDs in the version 3 annotation of the *P. tricornutum* genome.

*Phaeodactylum* plastid-related processes, established from previous studies (Ait-Mohamed et al., 2020) were divided into five functional groups (**Supplementary Table S3**): photosynthesis-related (relating either to light acquisition, photosynthetic electron transport, chlorophyll or carotenoid biosynthesis), amino acid-related (lysine, chorismate, branched-chain amino acid and cysteine synthesis; alongside Fe-S cluster synthesis, as a necessary precursor to cysteine synthesis, and aminoacyl-tRNA activation as necessary for protein synthesis), biogenesis-related (ribosomal proteins, protein import, and DNA-associated proteins), carbon-related (carbohydrate transport, Calvin cycle and glycolysis/gluconeogenesis pathways) and lipid-related (fatty acid synthesis and lipid head group exchange) (**Figure 2**). 22 transporters were found to be associated with all five major processes considered, suggesting that they are likely to have central roles in diatom plastid metabolism (**Figure 2**). Other transporters showed associations only with specific pathways. For example, J46742 and J34949 were found to only be associated with amino acid-related pathways; J32535, J36726, J36253, J44665 and J4286 were associated only with photosynthesis-related pathways; EG00042, J32491, J46051, J43785 were associated only with biogenesis-related functions; and EG02514 was associated only with fatty acid/lipid related functions. No transporters were found to be associated exclusively with carbon-related functions (**Figure 2**), reflecting the importance of central carbon metabolism for other plastid metabolic processes (e.g., amino acid and lipid biosynthesis) (Nonoyama et al., 2019; Ait-Mohamed et al., 2020).

### Identification of *P. tricornutum* plastid transporters potentially associated with mitochondrial crosstalk

The intricate metabolic connections between diatom plastids and mitochondria (Prihoda et al., 2012; Bailleul et al., 2015; Uwizeye et al., 2020) likely depend on transporters that transfer metabolites either between the two organelles, or with adjacent compartments such as the peroxisome (Shai et al., 2016; Dorrell et al., 2017; Mix et al., 2018). In order to find potential plastid transporters that may be related to this cross-talk, the average Pearson correlation coefficient values were calculated between 66 plastid transporters and 34 genes from the mitochondria genome, for which equivalent relative fold-expression change data were present in published microarray data (Oudot-Le Secq et al.; Ashworth et al., 2016).

**Figure 3:**
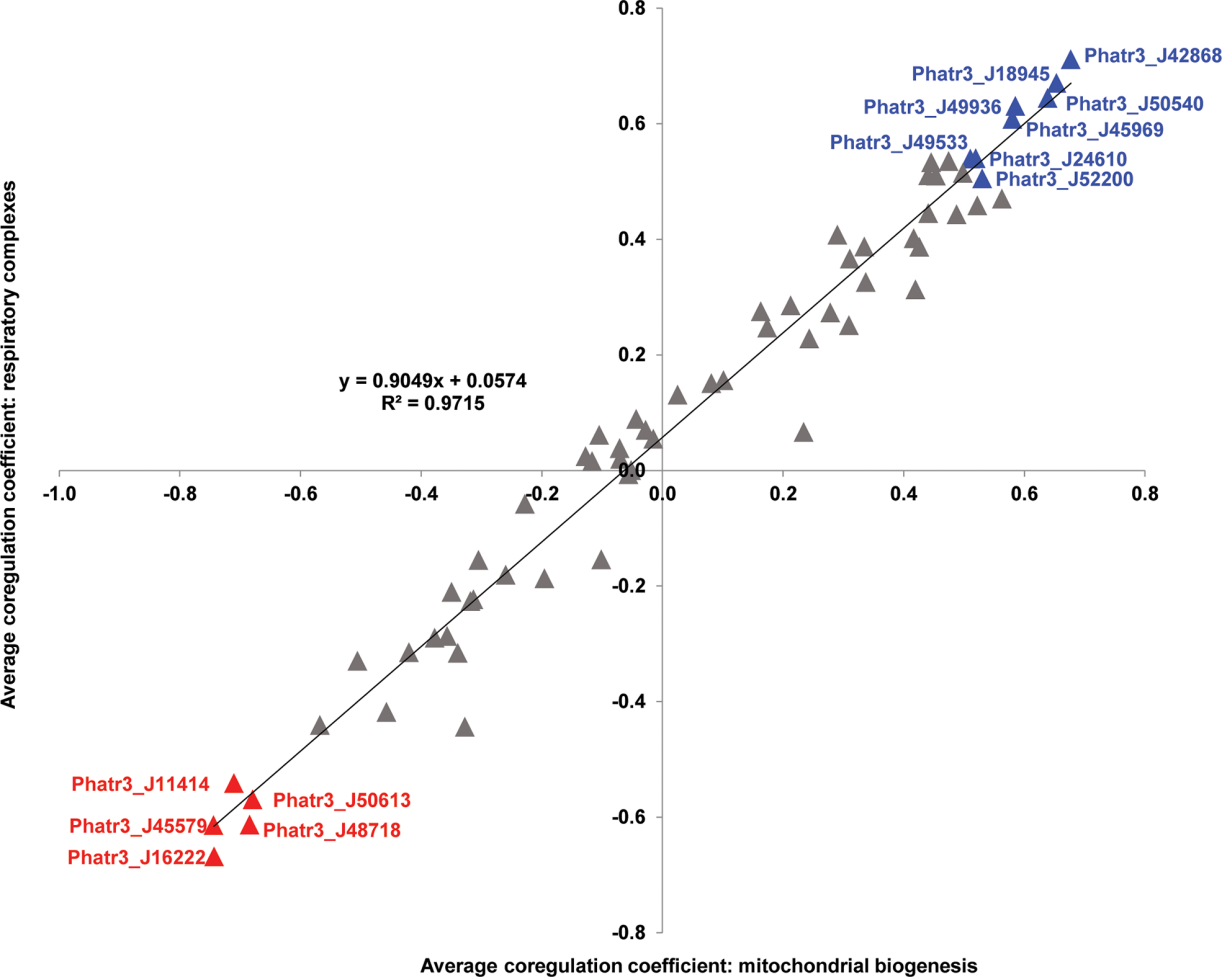
Scatterplot of Pearson correlation coefficients between plastid transporters and mitochondria-encoded genes. Horizontal axes correspond to average correlation coefficients calculated between *Phaeodactylum* transporter genes and mitochondrial genes involved in biogenesis (encoding ribosomal and translocase proteins), and vertical axes correspond to average correlation coefficients calculated between *Phaeodactylum* transporter genes and mitochondrial respiratory complex genes across 17 conditional groups in published microarray data studies (Ashworth et al, 2016; *ibid*). The strong positive correlation coefficient between both axes (*t*-test P < 10^-05^) indicates strong overall reproducibility of correlation, independent of mitochondrial gene function. Eight transporter genes that recover average correlation coefficients > 0.5 and five that recover average correlation coefficients < - 0.5 for both axes are labelled in blue and red text, respectively.

A total of 37 plastid transporters were found to have an average mitochondrial Pearson correlation > 0, and eight plastid transporters showed average values > 0.5, considering both correlation to mitochondrial respiratory complex and biogenesis genes (**Figure 3**). Only three plastid transporters (NTT1 ATP/ADP transporter J49533, triose phosphate transporter J24610, MFS transporter J18945) that showed Pearson correlation values > 0.5 retrieved BLASTp homologues with E-value ≤ 1e-05 against the *A. thaliana* genome (**Figure 3**; **Supplementary Table S2**). The remaining five had no obvious homology to *A. thaliana* proteins, suggesting that many of the transporters implicated in plastid-mitochondria crosstalk may be unique to diatoms, or at least not conserved with plants.

The strongly mitochondrially co-regulated transporters included four containing the MFS domain (J42868, J18945, J49533, J49936), and a triose phosphate transporter (J24610) (**Figure 3**; **Supplementary Table S2**). These results underline the probable roles of carbon metabolism in mediating diatom plastid-mitochondria interactions (Broddrick et al., 2019). The majority of these strongly mitochondrially co-regulated plastid transporters seem to be associated with more than one fundamental category in *P. tricornutum* metabolism, with five of them (J50540, J18945, J45969, J49936, J24610) linked to all five pathway categories (**Figures 2**, **3**). None of the remaining strongly mitochondrially co-regulated transporters showed functional associations with lipid-related metabolism (**Figures 2**, **3**), suggesting that lipids play more limited roles in diatom mitochondria-plastid crosstalk.

### Identification of diatom-specific plastid transporters from multispecies sequence datasets

Given the limited conservation observed with *A. thaliana*, we wished to understand the deeper evolutionary conservation and gene transfer events underpinning the origins of *P. tricornutum* plastid transporters. First, to assess the immediate evolutionary conservation of each transporter, we identified homologous sequences of all 70 P*. tricornutum* plastid transporters by RbH amongst a previously assembled composite library of more than two hundred species (Nonoyama et al., 2019; Dorrell et al., 2021) sampled from across stramenopiles, the broader group of organisms including diatoms. The composite library was further divided into three groups: diatoms (106 species), other members of the ochrophytes that contain plastids (94 species) and are predominantly photosynthetic, and aplastidic groups (23 species) that lack functional plastids. The composite library was further enriched with members of the cryptomonads (27 species) and haptophytes (37 species) which, like the photosynthetic members of the stramenopiles, have plastids derived from the secondary endosymbiosis of red algae and may share a common origin with the ochrophyte plastid, and are surrounded by four membranes (Dorrell and Smith, 2011). As each of these lineages possess similar plastid structures, plastid-targeted proteins can be predicted for them *in silico* using common tools HECTAR (Gschloessl et al., 2008) and ASAFind (Gruber et al., 2015), and RbH searches were also performed explicitly for proteins with inferred plastid-targeted sequences in each species.

**Figure 4:**
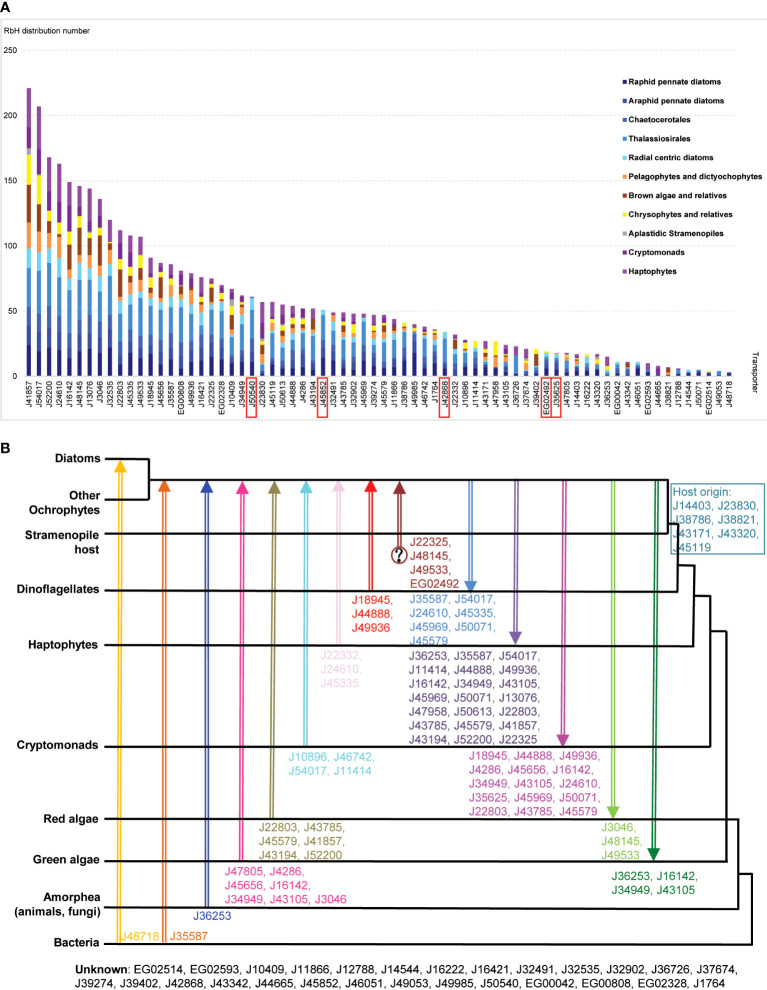
Evolutionary relationships between *P. tricornutum* plastid transporters. **(A)** Bar chart of the numbers of *P. tricornutum* plastid transporters that retrieved RbH over at least two species from 11 taxonomic groups (Materials and Methods; **Supplementary Table S4**). Taxonomic categories are grouped as diatoms (blue); other ochrophytes (yellow/orange); aplastidic stramenopiles (grey) or other lineages with secondary red plastids surrounded by four membranes (purple). Transporter genes inferred to be conserved across diatoms (present in >1 species in all diatom taxonomic categories considered) but not found elsewhere (present in < 4 non-diatom species, and no more than two in any one non-diatom taxonomic category) are labelled with red rectangles. **(B)** Phylogram of *P. tricornutum* transporters based on single-gene trees for each transporter with a taxonomically exhaustive reference dataset, per a prior phylogenomic study (Dorrell et al., 2021). Different gene transfer events between different taxonomic groups are shown by arrow colour and orientation, with transporter predicted to have undergone this gene transfer event are shown next to each arrow. Transporter genes inferred to have been vertically inherited from the aplastidic stramenopile host are displayed in the blue box, and transporters with unknown origins are shown at the bottom.

Unsurprisingly, given their evolutionary proximity, raphid pennate diatoms (other than *P. tricornutum*) were found to possess the greatest number (63) of homologues of *P. tricornutum* plastid transporters (**Figure 4A**); followed by other diatoms within the Thalassiosirales, Chaetocerotales, araphid pennate and radial centric lineages, for which 48-58 transporter homologues were detected (**Figure 4A**). The next highest numbers (40-44) of transporters with homologues were found in pelagophyte/dictyochophyte, pinguiophyte/chrysophyte, and haptophyte groups (**Figure 4A**). Fewer transporters were inferred to have homologues in ochrophytes within the PX/raphidophytes (33) and cryptomonads (31), and only five were found to have homologues in plastid-lacking stramenopiles (**Figure 4A**). As this last group is projected to have never acquired a red algal plastid (Dorrell et al., 2017; Stiller et al., 2009), this likely indicates that the majority of *P. tricornutum* plastid transporters have explicitly plastid-related evolutionary origins.

Using this approach, five transporters (J45852, J50540, J42868, EG02492, and J35625) were inferred to be conserved across, but unique to diatoms (**Figure 4A**). None of these five diatom unique transporters retrieved homologues in the *A. thaliana* genome (**Figure 1A**; **Supplementary Table S2**), while two (J42868: MFS domain transporter; J50540: plastid transport protein Tic110) showed the strongest and third strongest positive transcriptional correlations with the mitochondrial genome (**Figure 3**), suggesting possible diatom-specific effectors or regulators of plastid-mitochondria crosstalk. All of these diatom-specific transporters were transcriptionally co-regulated with at least four of the five functional categories of diatom plastid metabolism studied (**Figure 2**), suggesting that they play central roles in diatom cell metabolism.

Finally, we reanalyzed single-gene trees presented in a previously published phylogenetic study, which considered all transfer events, including genes received from and donated into other lineages, visible in the *P. tricornutum* genome (Dorrell et al., 2021) and extending to the last common ochrophyte ancestor. The results are presented in **Figure 4B** and **Supplementary Table S4**. Six transporter genes (J22803, J43785, J45579, J41857, J43194, J52200) were predicted to have arisen *via* transfers from red algae into ochrophytes (**Figure 4B**). A further ten transporters were predicted to be transferred from algae with secondary red plastids (cryptomonads, haptophytes or dinoflagellates) into the ochrophyte common ancestor, reflecting the red algal origin of the *P. tricornutum* plastid (**Figure 4B**). Seven transporter genes (J47805, J4286, J45656, J16142, J34949, J43105, J3046) likely originated from green algae into ochrophytes, while two transporters (J48718: a permease, and J35587: a multidrug and toxic compound extrusion family protein) were inferred to have arisen from horizontal transfers from bacteria into, respectively, diatoms specifically, or all ochrophyte lineages. These latter transporter groups reflect the chimeric composition of the diatom plastid proteome, which is supported by nucleus-encoded and plastid-targeted proteins of red, green, host and bacterial origin (Oborník and Green, 2005; Nonoyama et al., 2019).

### Environmental correlations with abundance of diatom plastid transporters in *Tara* Oceans data

We wished to identify the expression trends of diatom plastid transporters under different environmental factors, and find transporters that show unique trends with environmental variation. We extracted meta-transcriptome homologues of *P. tricornutum* plastid transporters from version 1 of the *Tara* Oceans Expedition Ocean Gene Atlas (Villar et al., 2018), and identified sequences that corresponded specifically to diatom plastid-targeted proteins by phylogenetic approaches (**Supplementary Table S5**). A total of 65 of the 70 P*. tricornutum* transporters were found to possess multiple environmental homologues, and were selected for subsequent quantitative analysis of meta-transcriptome (MetaT) and meta-genomes (MetaG) abundance against sampled environmental variables at each depth and size fraction. Krona Plots concerning the nearest homologue from cultured species of all phylogenetically identified diatom transporter meta-gene are provided in **Supplementary Figure S1**. Confirming the accuracy of the phylogenetic identification approach employed, the majority (90%) of these homologues correspond to stramenopiles, and 97% of these stramenopiles belong to diatoms. None were precisely annotated as *P. tricornutum*, reflecting its rarity in environmental samples (Malviya et al., 2016).

**Figure 5:**
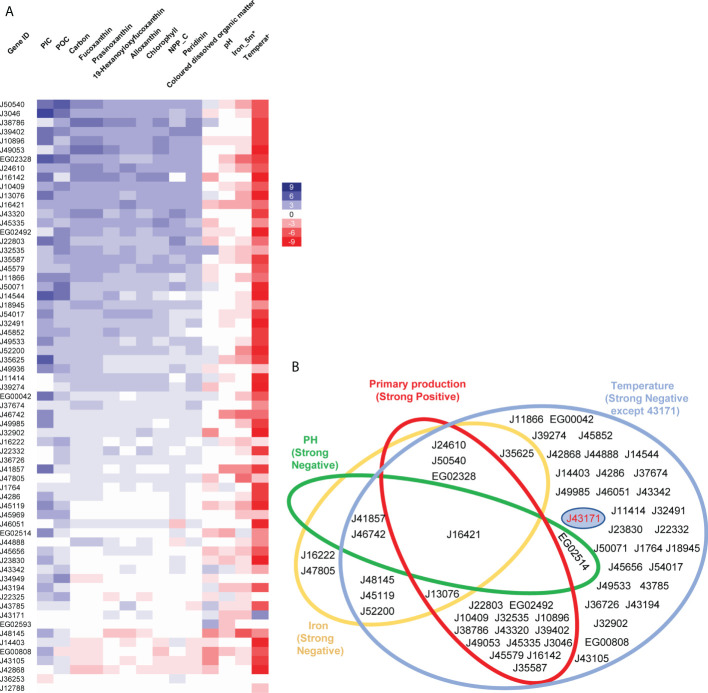
Relationships between abundance of diatom plastid transporters and environmental parameters. **(A)** Heatmap showing relationships of environmental factors with 65 P*. tricornutum* transporter homologues in *Tara* Oceans meta-genome (MetaG) and meta-transcriptome (MetaT) datasets. The calculations for each transporter were performed using data from all *Tara* Oceans sampling stations in version 1; with independent calculations for each combination of size fractions (0.8 to 5, 5 to 20, 20 to 180, 180 to to 2000; and 0.8 to 2000 μm) and depths (DCM, deep chlorophyll maximum layer; SRF, upper layer zone) performed. Heatmap rows correspond to transporter gene IDs in *P. tricornutum*, columns represent different environmental parameters. Blue denotes positive relationships between transporters and environmental factors, red denotes negative relationships, with shade proportional to the number of depth and size fraction combinations (maximum 10) showing a significant (P< 0.05) Pearson correlation. Aggregate values are shown, that is to say that a significant positive correlation (score +1) in one depth and size fraction combination, and a significant negative correlation (score -1) in another would equate to an aggregate score of 0. POC: Particulate Organic Carbon (µg/kg), PIC: Particulate Inorganic Carbon (mol/m^3^), Carbon: Carbon Total (µmol/kg), NCC_C: Net primary production of carbon (mg/m^2^/day). **(B)** Plastid transporters showing strong relationships (P< 0.05 in at least three different combinations of depths and size fractions; for merged primary production, significant correlations in ≥ 3 depth and size fractions combinations for ≥38 of the ten selected parameters) with four environmental parameters, these being: temperature (blue, negative related except J43171), iron (yellow, negative related), pH (green, negative related) and primary production related parameters (red, positive related, merged by Chlorophyll c3 (mg/m^3^), Peridinin (mg/m^3^), Fucoxanthin (mg/m^3^), Prasinoxanthin (mg/m^3^), 19-Hexanoyloxyfucoxanthin (mg/m^3^), Alloxanthin (mg/m^3^), NCC (mg/m^2^/day), PIC (mol/m^3^), POC (µg/kg), Carbon Total (µmol/kg). J43171 uniquely displays a positive relationship with temperature and is separately displayed by a blue circle in the temperature circle. Transporters are shown as gene IDs from *P. tricornutum*.

Many of the diatom plastid transporters showed similar correlations to different environmental factors (**Figure 5A**), e.g., positive transcriptional correlations with primary production-related parameters (pigments, net primary production, particulate inorganic, organic and total carbon), and negative correlations with iron, pH, and temperature (**Figure 5A**). These trends likely reflect diatom environmental preferences for high-latitude, nutrient-rich but iron-limited, and highly productive environments (Malviya et al., 2016; Benoiston et al., 2017; Nonoyama et al., 2019; Young and Schmidt, 2020).

Other environmental factors showed more variable relationships with diatom plastid transporters. For example, colored dissolved organic matter (CDOM), showed a strong positive correlation (0< P< 0.05 observed between a parameter and transporter expression abundance across ≥ three combinations of depth and size fraction) with two transporters, and strong negative correlations (-0.05< P< 0 observed between a parameter and transporter expression abundance across ≥ three combinations of depth and size fraction) with five transporters (**Figure 5A**; **Supplementary Table S5**). The transporters that showed strong positive environmental correlations to CDOM (EG02593, J32535) appeared to have more specific functional relationships in our Venn diagram of *P. tricornutum* cell metabolism, while the transporters that showed strong negative relationships to CDOM (EG00808, J32902, J16421, J43105, J48145) were found to be associated with multiple *P. tricornutum* plastid metabolism pathways (**Figures 2**, **5**). These relationships may identify novel biomarkers of diatom plastid functional health, and of carbon export from the ocean surface layer (Guidi et al., 2016; Sunagawa et al., 2020).

### Identification of specific diatom plastid transporters linked to individual marine processes

Despite these global biases, we noted distinctive environmental expression trends for different diatom plastid transporters. We summarize the significant relationships for each transporter in **Figure 5B**, grouping the environmental parameters into four functional categories: temperature, iron, pH and ten merged primary production related parameters (see Materials and Methods). No transporter was significantly correlated with all of the environmental parameters studied (**Figure 5B**), indicating probable physiological partitioning of transporter functions in relation to different environmental factors. The two transporter genes showing the strongest positive and negative relationships to primary production were, respectively, J50540 (plastid transport protein Tic110) and J42868 (MFS domain transporter) (**Figure 5A**; **Supplementary Table S5**). These two genes were also among the two most strongly correlated plastid transporters to the *P. tricornutum* mitochondrial genome (**Figure 3**), underpinning the importance for plastid-mitochondrial crosstalk in supporting diatom photosynthetic activity, and potentially even in antagonistic interactions with primary production. Other plastid transporter genes showed strong negative relationships with pH (mitochondrial carrier protein J46742 and ABC transporter J41857) and iron (J48145, an EamA-like/drug-metabolite transporter) providing potential new biomarkers for understanding diatom responses to ocean acidification and biological competition (**Figure 5A**; **Supplementary Table S5**).

One transporter, J43171, encoding an ion channel protein with an EF-hand calcium-binding motif, uniquely showed a weak positive relationship with temperature in the *Tara* Oceans data (**Figures 5A**, **B**). This transporter was found to be associated to plastidial carbon, photosynthesis and biogenesis-related pathways in *P. tricornutum* transcriptome data (**Figure 2**) albeit with limited co-regulation with the mitochondria (**Supplementary Table S3**); showed a sporadic distribution across photosynthetic stramenopile and haptophyte transcriptomes (**Figure 4**); and lacks any observable *A. thaliana* homologue (**Figure 1**). *Tara* homologue abundance distribution maps of transporter J43171 and J50540 (as a representative transporter strongly positively correlated to marine primary production) are shown in **Supplementary Figure S2** and **Supplementary Figure S3**. The more specific functions of these transporters, particularly their functions in relation to oceanic temperature and photosynthetic activity remain to be confirmed e.g., through the phenotyping of mutant lines in transformable model species such as *Phaeodactylum*.

## Discussion

Although plastid transporters play important roles in energy supply and many other physiological and biochemical reactions in eukaryotic photosynthetic organisms, existing research has largely focused on those found in higher plants, while comprehensive analyses of plastid transporters in eukaryotic algae are still relatively rare (Marchand et al., 2018; Marchand et al., 2020). Here, we profile diverse bioinformatic datasets constructed around the model diatom *P. tricornutum*, alongside environmental sequence data from *Tara* Oceans, to close the knowledge gap concerning the plastid transporters associated with diatoms, and other related algal groups with four-membranes surrounded plastids derived from the secondary endosymbiosis of red algae (e.g., stramenopiles, cryptomonads, and haptophytes). Complementing more detailed studies from other groups on specific proteins (Marchand et al., 2018; Marchand et al., 2020), our data provide holistic insights into the metabolic, evolutionary and environmental functions of diatom plastid transporters.

We note that the transporters identified in our approach (i.e., *via* the presence of a bipartite cleavable targeting sequence, Gruber et al., 2015) may localize to one of multiple membranes (i.e., the plastidial inner or outer envelopes, and the periplastidial membrane; **Figure 1C**, **Figure 1D**). The precise resolution of membrane localization and orientation of specific diatom plastid transporters awaits detailed functional characterization, e.g. *via* self-assembling GFP or cross-linking approaches (Moog et al., 2015; Moog et al., 2020). We also note that there are likely to be further transporters necessary for diatom plastidial function not detected by this approach (e.g., transporters targeted to the chloroplast endoplasmic reticulum, and transporters targeted to the plastid *via* non-conventional pathways that do not utilize conventional plastid targeting sequences). These additional transporters may be best revealed by targeted proteomics either of isolated diatom chloroplasts (Schober et al., 2019), or quantitative proteomics of whole-cell separations (e.g., LOPIT, Mulvey et al., 2017 and Barylyuk et al., 2020).

Our analyses reveal similarities and differences in the transporter profiles of diatom and plant plastids (**Figure 1A**, **Figure 1B**), reflecting their deep evolutionary divergence. We found clear homologue equivalents of key *A. thaliana* ion, nucleotide and sugar plastid transporters in the *P. tricornutum* genome (**Figure 1B**). Our data corroborates possible differences in substrate specificity between homologous *A. thaliana* and *P. tricornutum* plastid metabolite transporters (**Figure 1B**), e.g., the triose phosphate transporters J24610 and J54017, which appear to be nearest equivalents of *A. thaliana* TPT, GPT, XPT and PPT transporters (**Figure 1B**). It has been reported that TPT homologues in other red alga-derived composite plastids, e.g. those of the non-photosynthetic diatom *Nitzschia putrida*, may have bifunctional TP and PEP transport capabilities (Moog et al., 2020), although the case of in *P. tricornutum* this awaits specific functional characterization, e.g., by electrophysiology or *via* micelle incorporation and *in vitro* assays (Shukla et al., 2017; Wright et al., 2018; Nielsen et al., 2019). Alongside this, we identify clear differences in plant and diatom plastid transporter architecture, including at least six unique to diatoms and not found in other stramenopile, cryptomonad or haptophyte groups (**Figure 4**). The exact evolutionary origins of these diatom-specific plastid transporters will be best revealed by detailed phylogenies of each protein, incorporating densely sampled phylogenetic reference datasets and *in silico* localization predictions, to identify their probable histories of gene transfer, duplication and re-localization within the diatom cell.

The transporter architecture of the diatom plastid may underpin its unique metabolic potential (Prihoda et al., 2012; Marchand et al., 2018; Nonoyama et al., 2019). Previously, for example, Bailleul et al. have proposed that import of mitochondrial ATP and export of plastid NADPH facilitates diatom photo-acclimation to high light and post-illumination conditions (Bailleul et al., 2015). Our data highlight plastid transporters that show strong transcriptional co-regulation with the mitochondrial genome (**Figure 3**), and may be involved in plastid-to-mitochondria metabolite exchange. We also identify by *in silico* prediction at least five transporters that are potentially dual-targeted to the plastids and mitochondria (**Supplementary Table S2**). Finally, we note that at least two of the plastid transporters that show the strongest transcriptional coordination to the *P. tricornutum* mitochondria (J42868: MFS domain transporter; J50540: plastid transport protein Tic110) also had strong correlations to primary production in *Tara* Oceans data (**Figures 3**, **5**), underlining the importance of plastid/mitochondria metabolite exchange for diatom fitness in the environment. The localizations and functions of transporters putatively involved in *P. tricornutum* plastid/mitochondria crosstalk may be best explored by experimental localization, and functional characterisation of mutant lines.

Our environmental data provide insights into which plastid transporters may direct diatom sensitivity to anthropogenic environmental impacts. Rising seawater temperatures (i.e., caused by global warming) and pH decreases (ocean acidification), will most likely individually or synergistically influence diatom photosynthesis, respiration (Goldman et al., 2017; Shi et al., 2019) and primary metabolism (Bermúdez et al., 2015; Novak et al., 2019); although with rising temperatures predicted to have greater overall impact on diatom biomass and photosynthesis than acidification (Sommer et al., 2015; Zhong et al., 2021). Concordant with this, our *Tara* Oceans-based analysis of environmental factors indicates that most diatom plastid transporters showed negative relationships with temperature but no clear response to pH (**Figure 5**).

We see contrasting relationships between the expression parameters of different diatom plastid transporters with CDOM. The physiological links between transporter expression and this environmental parameter, which is influenced by both primary production, photodegradation and decomposition (Zhong and Wang, 2009; Zou et al., 2012), and in turn is likely to influence algal photosynthesis and nutrient uptake (White et al., 2003; Helms et al., 2013) alongside algal-bacterial interactions (Klug, 2005), are more complex, and may best be explored by mesocosm-related environmental expression measurements. Finally, the uniquely temperature-associated transporter J43171 (**Figure 5B**) may provide new insights into diatom thermal adaptation and climate resilience, which may be explored either by cellular (mutant phenotyping, experimental evolution) or environmental approaches (e.g., sequencing of diatoms isolated from subtropical and warm-water habitats).

In conclusion, our holistic study is significant to broader studies of diatom plastid metabolic, evolutionary and environmental functions, allowing the pinpointing of targets linked to key physiological and environmental processes for functional and experimental characterization.

## Data availability statement

The datasets presented in this study can be found in online repositories. The names of the repository/repositories and accession number(s) can be found below: osf.io repository https://osf.io/89vm3/ in the folder “Transporters”.

## Author contributions

SL and RGD conceived the project and designed research; SL, MS, and RGD performed research; SL wrote the paper; CB, RGD, and GF contributed to funding acquisition; all authors contributed to the article and approved the submitted version.

## Funding

This work was supported by grants from the Chinese Scholarship Council (awarded 2019-2023, scholarship file No. 201904910555) to SL, a CNRS Momentum Fellowship (awarded 2019-2021), and an ANR JCJC (ANR-21-CE02-0014-01) to RGD, an ANR Collaborative grant (ANR-19-CE20-0020) of France to CB, and a European Research Council Advanced Grant (Chloro-mito, Grant No. 833184) to GF.

## Acknowledgments

SL acknowledges a Chinese Scholarship Council PhD student (awarded 2019-2023, scholarship file No. 201904910555). RD acknowledges a CNRS Momentum Fellowship (awarded 2019-2021) and an ANR t-ERC (ChloroMosaic). MS and GF acknowledge the support by the European Research Council (ERC) Chloro-mito (Grant No. 833184). CB acknowledges an ANR collaborative grant (Browncut, Grant No. ANR-19-CE20-0020). The authors thank Morgane Roquais and Juan Pierella Karlusich (IBENS) for assistance in the retrieval of Tara Oceans homologues for Phatr3_J43171. This is article # ABCDEF in the *Tara* Oceans series.

## Conflict of interest

The authors declare that the research was conducted in the absence of any commercial or financial relationships that could be construed as a potential conflict of interest.

## Publisher’s note

All claims expressed in this article are solely those of the authors and do not necessarily represent those of their affiliated organizations, or those of the publisher, the editors and the reviewers. Any product that may be evaluated in this article, or claim that may be made by its manufacturer, is not guaranteed or endorsed by the publisher.

## Supplementary material

Supplementary Table 1 List of *A. thaliana* plastid transporters inferred from ChloroKB, BLASTp outputs and localization predictions.

Supplementary Table 2 List of *P. tricornutum* plastid transporters inferred *via in silico* prediction, BLASTp outputs and localization predictions.

Supplementary Table 3 List of genes in *P. tricornutum* genome inferred to show transcriptional co-regulation, localization and evolutionary associations with plastid transporters, and their inferred metabolic and biological functions.

Supplementary Table 4 List probable evolutionary conservations of transporters inferred from phylogeny and RbH analyses.

Supplementary Table 5 Raw data for transporter environmental relationships.

Supplementary Figure 1 Pie chart of the taxonomic distribution of all identified diatom transporter meta-gene homologs.

Supplementary Figure 2
*Tara* homologue MetaT and MetaG abundance distribution maps of environmental homologues of the transporter J43171.

Supplementary Figure 3
*Tara* homologue MetaT and MetaG abundance distribution maps of environmental homologues of the transporter J50540.

Supplementary Data Sheet 1 Fasta format sequences of all 70 inferred *Phaeodactylum* transporter proteins used in this study.
