# Supplementary material for: A metabolic, phylogenomic and environmental atlas of diatom plastid transporters from the model species Phaeodactylum
Source: Front Plant Sci. 2022 Sep 22;13:950467. doi: 10.3389/fpls.2022.950467 (PMC9546453; doi:10.3389/fpls.2022.950467)

Taxonomic distribution of all identified diatom transporter meta-gene homologs

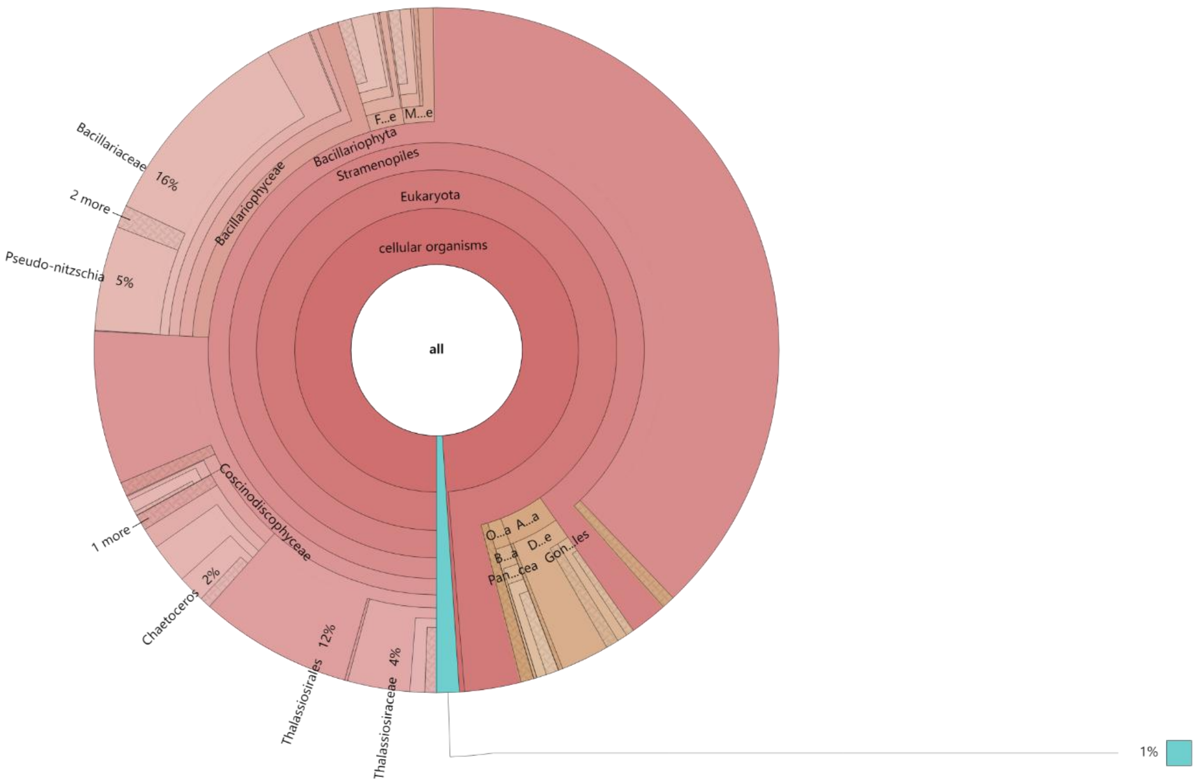

Taxonomic distribution details of Stramenopiles

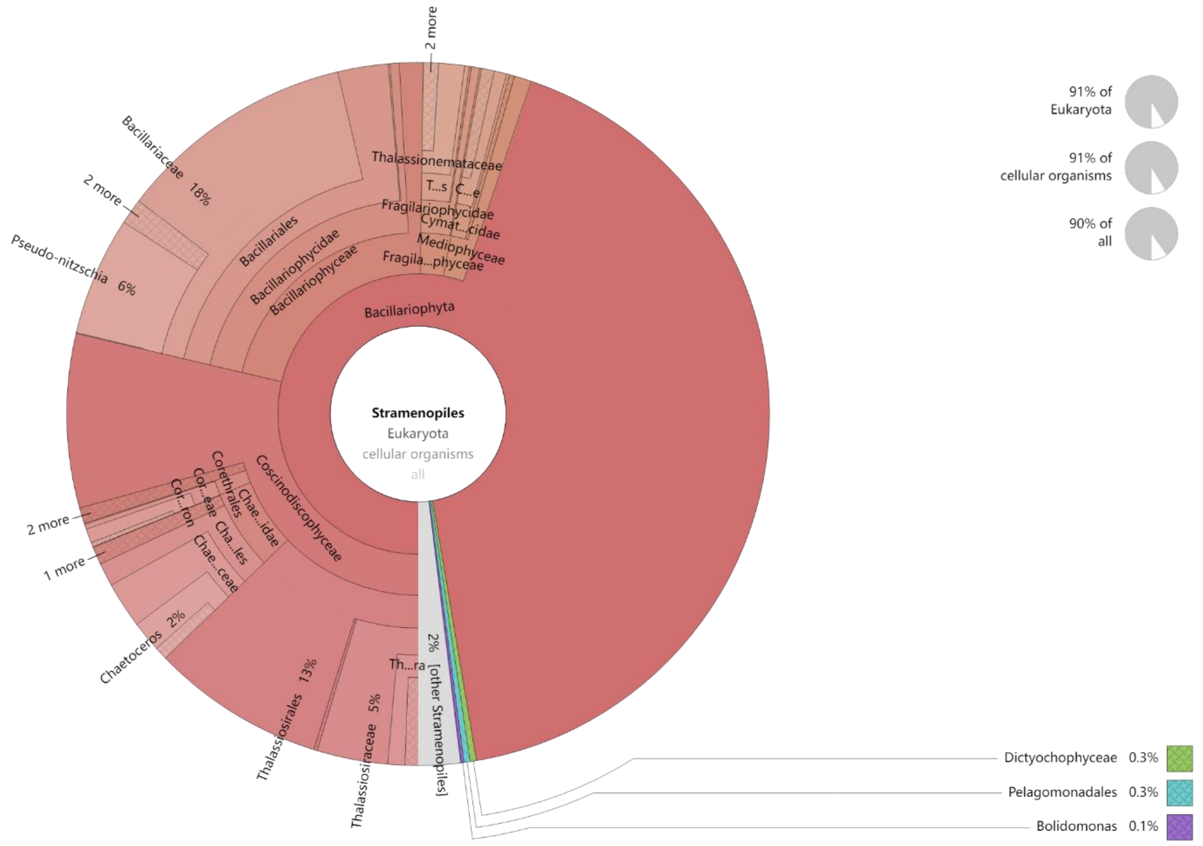

Fig. S1. Krona plots of the taxonomic annotations of Tara Oceans meta-gene phylogenetically assigned to being diatom plastid transporters.

Taxonomic distribution details of Diatoms

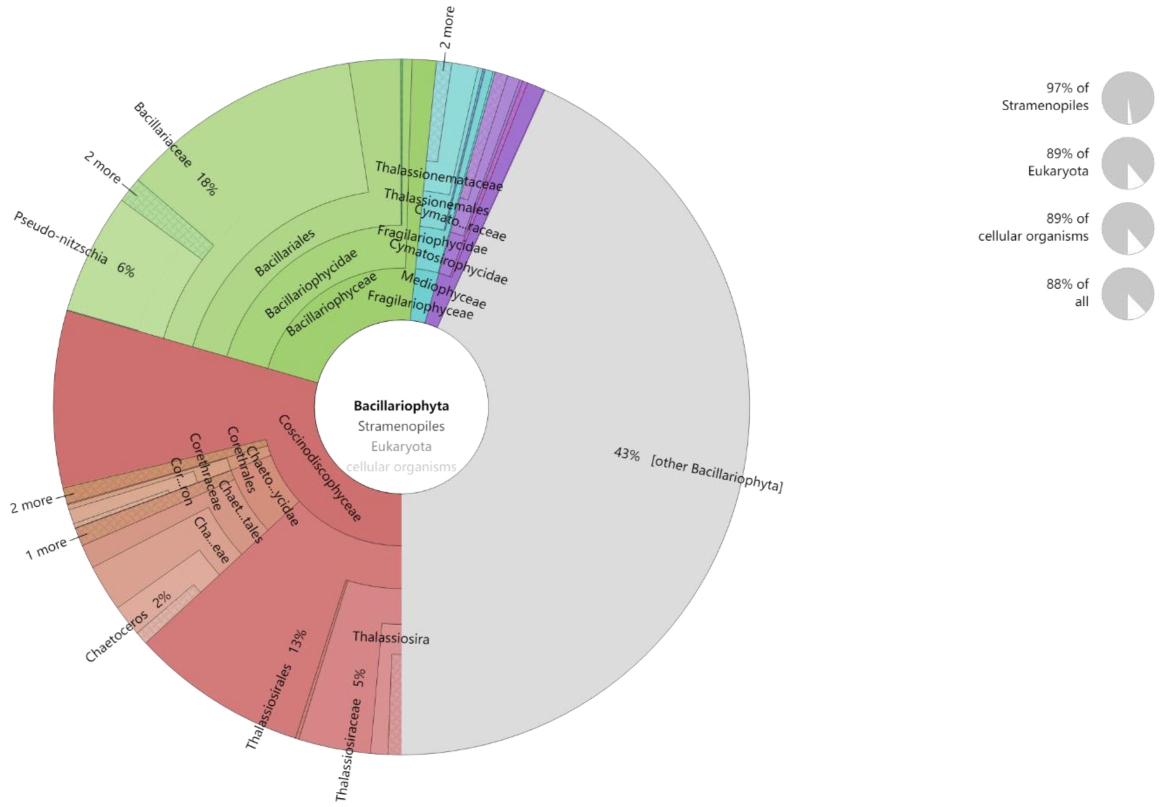

Supplement: Supplementary Figure 1 — Pie chart of the taxonomic distribution of all identified diatom transporter meta-gene homologs. [file Image_1.pdf]
