# Supplementary material for: A metabolic, phylogenomic and environmental atlas of diatom plastid transporters from the model species Phaeodactylum
Source: Front Plant Sci. 2022 Sep 22;13:950467. doi: 10.3389/fpls.2022.950467 (PMC9546453; doi:10.3389/fpls.2022.950467)
Supplement: Supplementary Figure 2 — Tara homologue MetaT and MetaG abundance distribution maps of environmental homologues of the transporter J43171. [file Image_2.pdf]

J43171-MetaT-DCM abundance distribution map

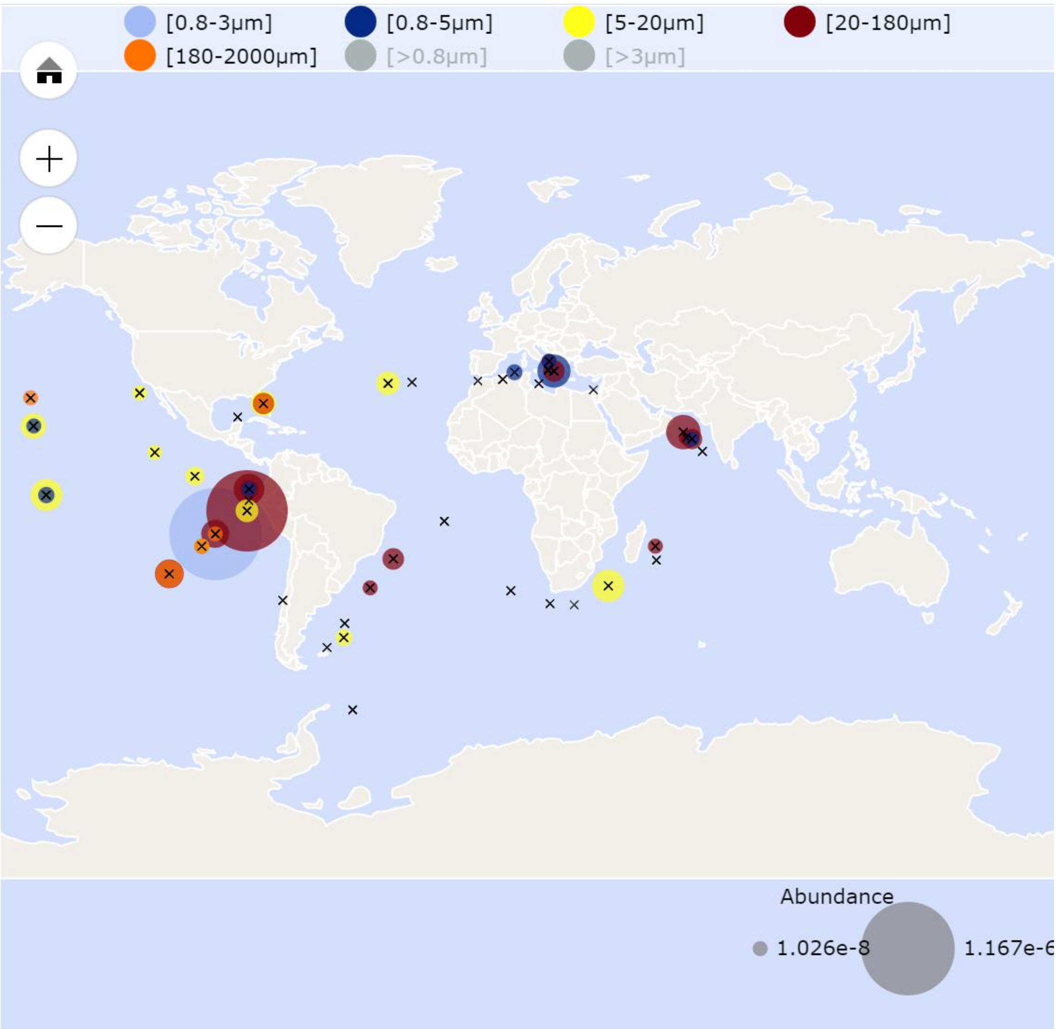

J43171-MetaT-SRF abundance distribution map

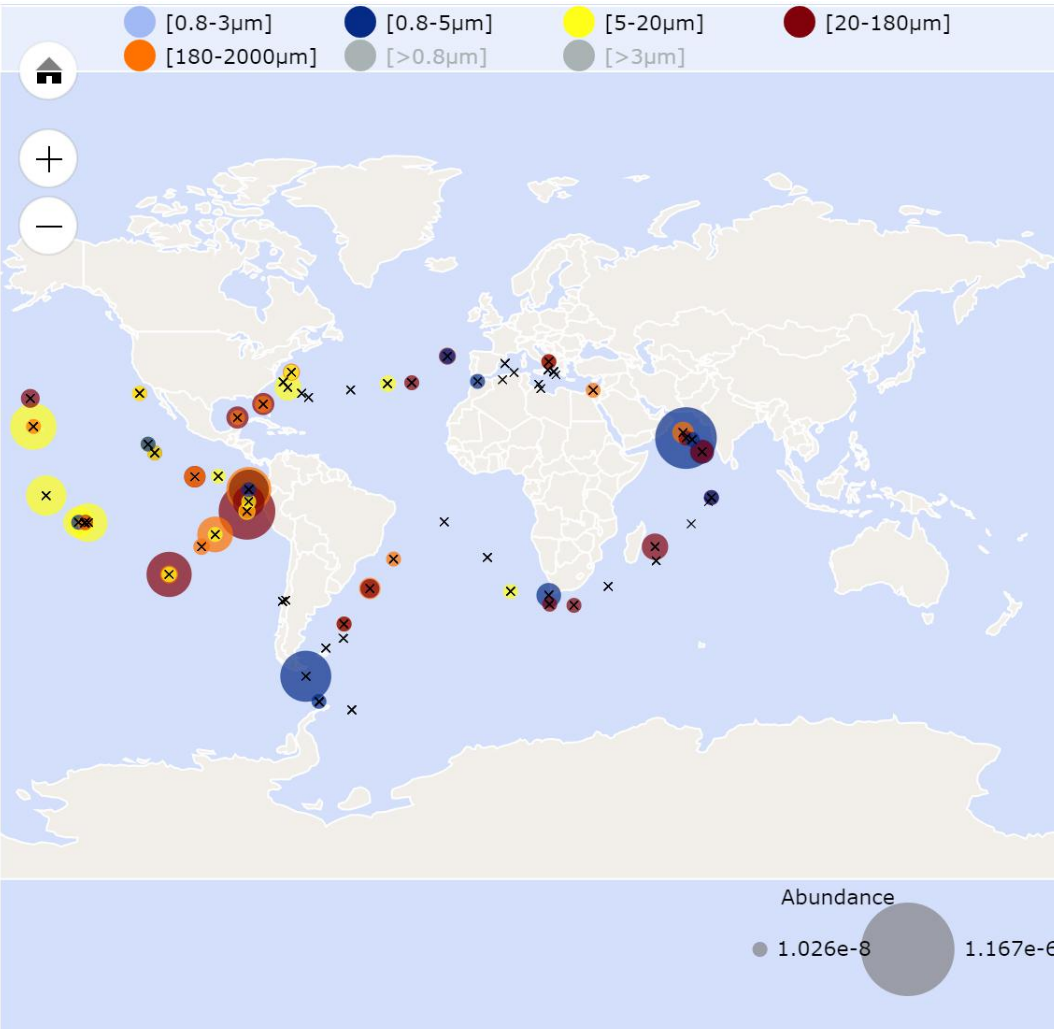

Relative MetaT abundances of phylogenetically identified diatom homologues of Phatr3_J43171 across DCM and surface depths for all size fractions from Version 1 Tara Oceans data.

J43171-MetaG-DCM abundance distribution map

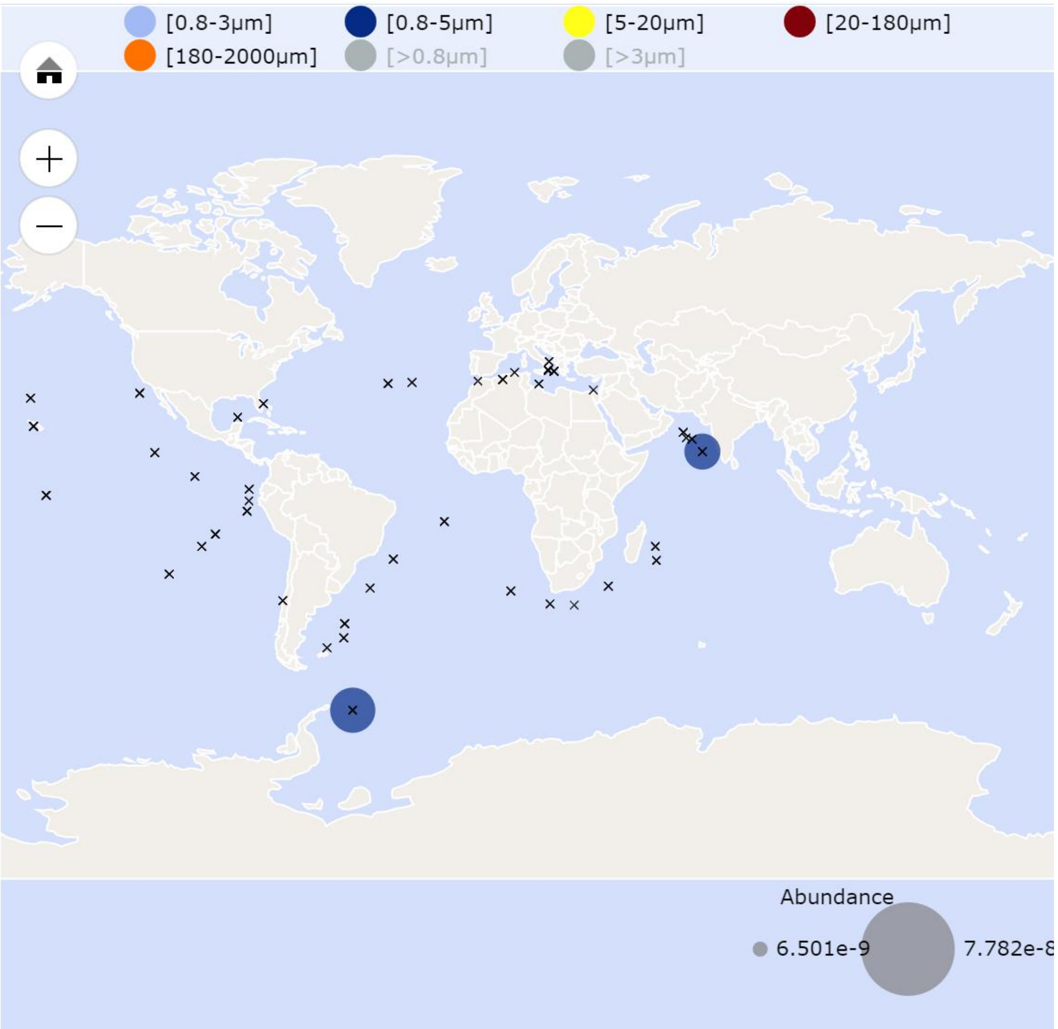

J43171-MetaG-SRF abundance distribution map

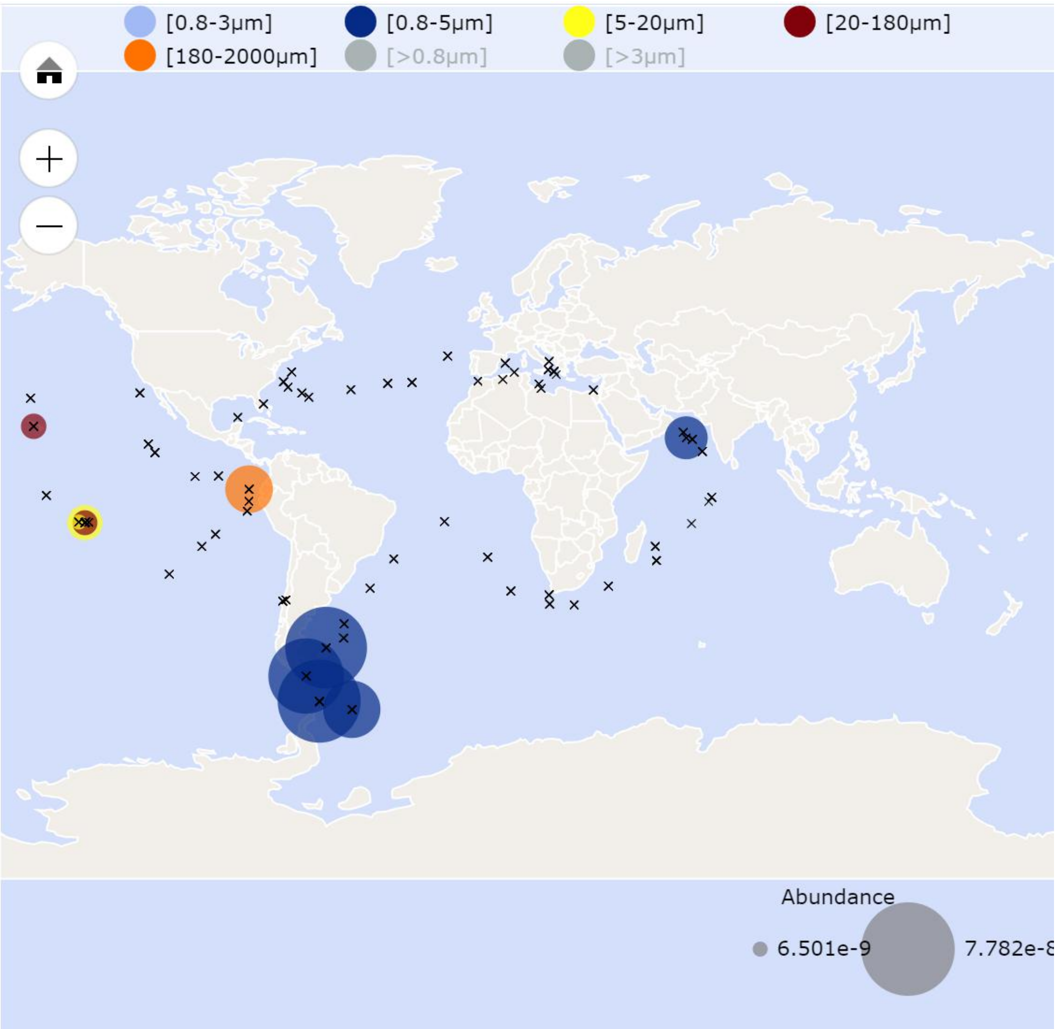

Relative MetaG abundances of phylogenetically identified diatom homologues of Phatr3_J43171 across DCM and surface depths for all size fractions from Version 1 Tara Oceans data.
