# Supplementary material for: A metabolic, phylogenomic and environmental atlas of diatom plastid transporters from the model species Phaeodactylum
Source: Front Plant Sci. 2022 Sep 22;13:950467. doi: 10.3389/fpls.2022.950467 (PMC9546453; doi:10.3389/fpls.2022.950467)

J50540-MetaT-DCM abundance distribution map

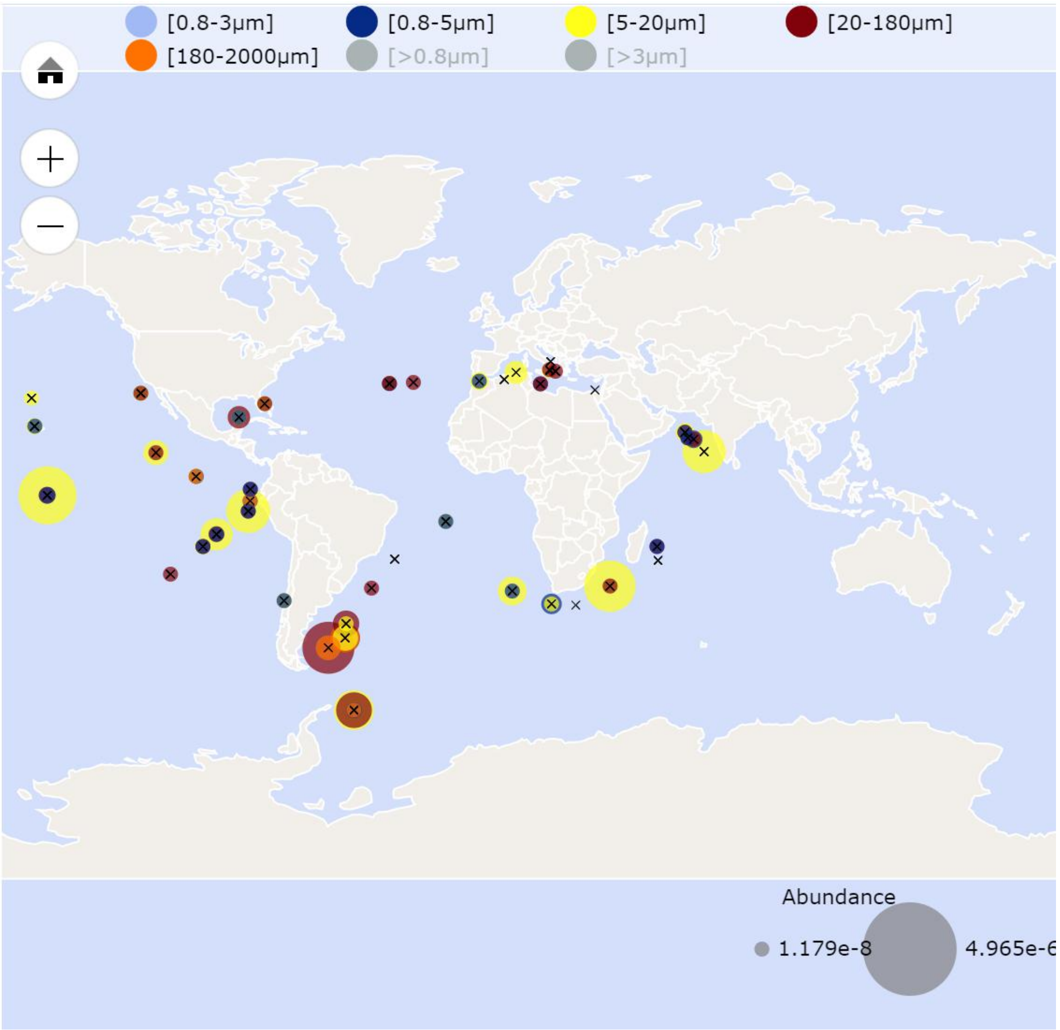

Relative MetaT abundances of phylogenetically identified diatom homologues of Phatr3_J50540 across DCM and surface depths for all size fractions from Version 1 Tara Oceans data.

J50540-MetaT-SRF abundance distribution map

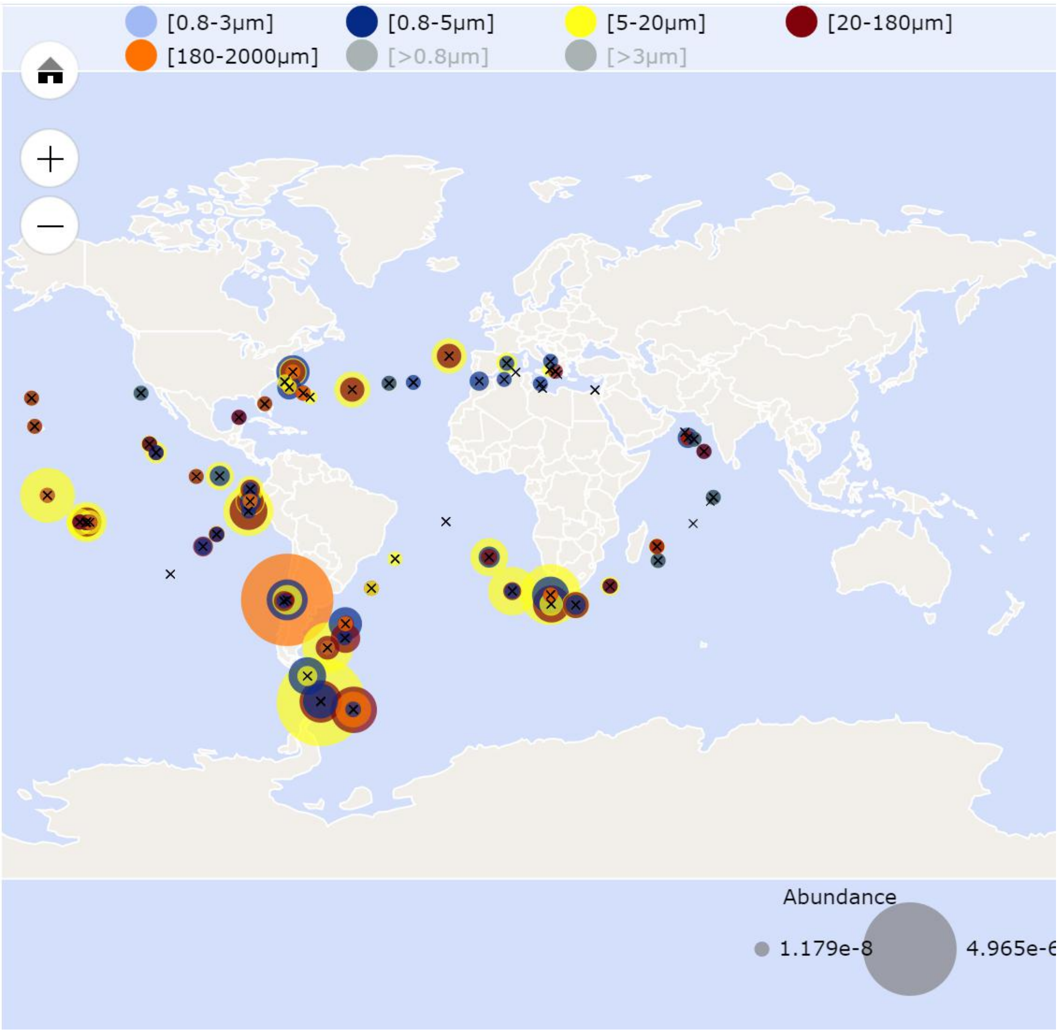

J50540-MetaG-DCM abundance distribution map

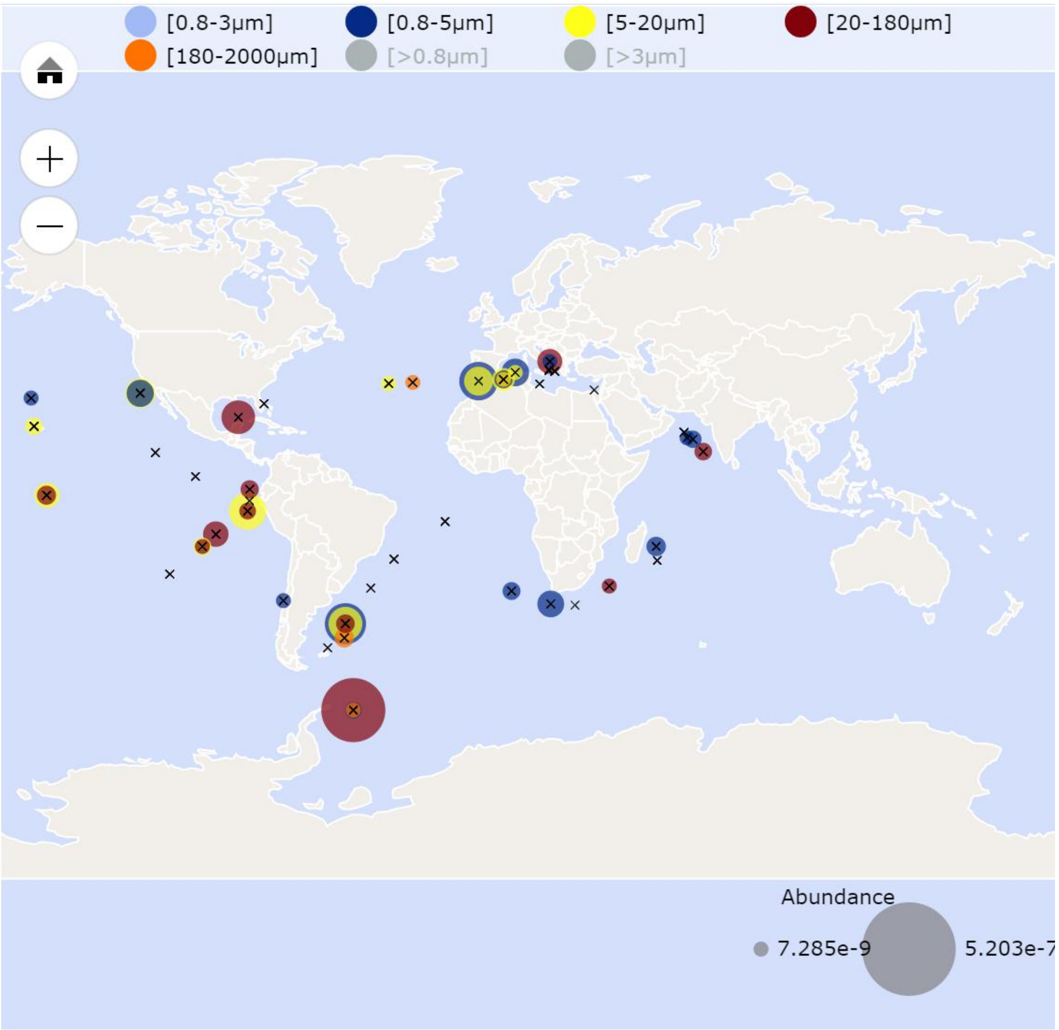

Relative MetaG abundances of phylogenetically identified diatom homologues of Phatr3_J50540 across DCM and surface depths for all size fractions from Version 1 Tara Oceans data.

J50540-MetaG-SRF abundance distribution map

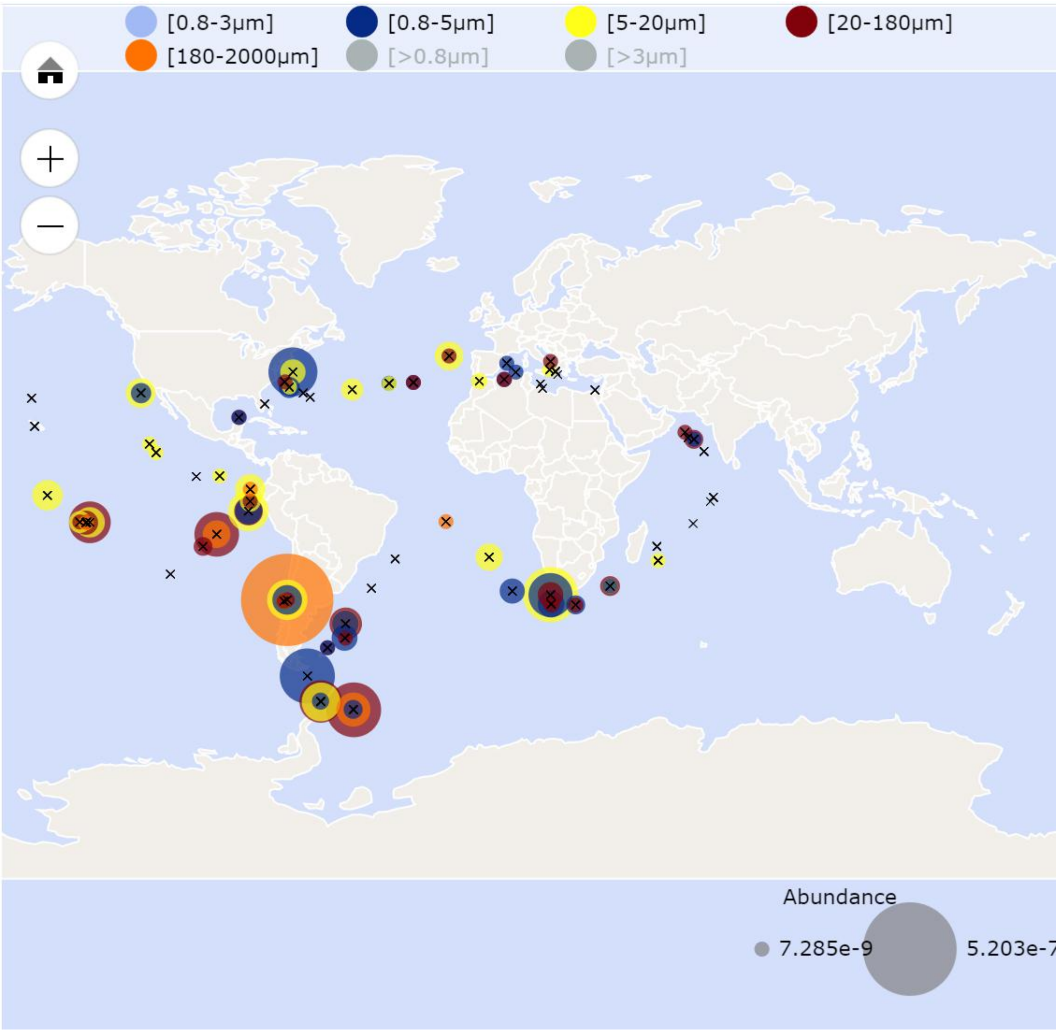

Supplement: Supplementary Figure 3 — Tara homologue MetaT and MetaG abundance distribution maps of environmental homologues of the transporter J50540. [file Image_3.pdf]
